# Dense genotyping of immune-related susceptibility loci reveals new insights into the genetics of psoriatic arthritis

**DOI:** 10.1038/ncomms7046

**Published:** 2015-02-05

**Authors:** John Bowes, Ashley Budu-Aggrey, Ulrike Huffmeier, Steffen Uebe, Kathryn Steel, Harry L. Hebert, Chris Wallace, Jonathan Massey, Ian N. Bruce, James Bluett, Marie Feletar, Ann W. Morgan, Helena Marzo-Ortega, Gary Donohoe, Derek W. Morris, Philip Helliwell, Anthony W. Ryan, David Kane, Richard B. Warren, Eleanor Korendowych, Gerd-Marie Alenius, Emiliano Giardina, Jonathan Packham, Ross McManus, Oliver FitzGerald, Neil McHugh, Matthew A. Brown, Pauline Ho, Frank Behrens, Harald Burkhardt, Andre Reis, Anne Barton

**Affiliations:** 1 Arthritis Research UK Centre for Genetics and Genomics, The University of Manchester, Manchester M13 9PT, UK; 2 NIHR Manchester Musculoskeletal Biomedical Research Unit, Central Manchester Foundation Trust and University of Manchester, Manchester Academy of Health Sciences, Manchester M13 9WU, UK; 3 Institute of Human Genetics, University of Erlangen-Nuremberg, Erlangen 91054, Germany; 4 The Dermatology Centre, Salford Royal NHS Foundation Trust, University of Manchester, Manchester Academic Health Science Centre, Manchester M6 8HD, UK; 5 JDRF/Wellcome Trust Diabetes and Inflammation Laboratory, Department of Medical Genetics, NIHR Cambridge Biomedical Research Centre, Cambridge Institute for Medical Research, University of Cambridge, Wellcome Trust/MRC Building, Cambridge Biomedical Campus, Cambridge CB2 0XY, UK; 6 Centre for Biostatistics, Institute of Population Health, The University of Manchester, Jean McFarlane Building, Oxford Road, Manchester M13 9PL, UK; 7 The Kellgren Centre for Rheumatology, Central Manchester Foundation Trust, NIHR Manchester Biomedical Research Centre, Manchester M13 9WL, UK; 8 Monash University, Melbourne, Victoria 3800, Australia; 9 NIHR-Leeds Musculoskeletal Biomedical Research Unit, Leeds Institute of Molecular Medicine, University of Leeds, Leeds LS7 4SA, UK; 10 CogGene Group, Discipline of Biochemistry and School of Psychology, National University of Ireland, Galway, Ireland; 11 Department of Clinical Medicine, Institute of Molecular Medicine, Trinity College Dublin, Dublin 8, Ireland; 12 Adelaide and Meath Hospital and Trinity College Dublin, Dublin 24, Ireland; 13 Royal National Hospital for Rheumatic Diseases and Department of Pharmacy and Pharmacology, University of Bath, Bath BA1 1RL, UK; 14 Department of Public Health and Clinical Medicine, Rheumatology, University Hospital, Umeå 901 87, Sweden; 15 Department of Biopathology, Centre of Excellence for Genomic Risk Assessment in Multifactorial and Complex Diseases, School of Medicine, University of Rome ‘Tor Vergata’ and Fondazione PTV ‘Policlinico Tor Vergata’, Rome 18-00173, Italy; 16 Rheumatology Department, Haywood Hospital, Health Services Research Unit, Institute of Science and Technology in Medicine, Keele University, Keele ST5 5BG, UK; 17 Department of Rheumatology, St. Vincent’s University Hospital, UCD School of Medicine and Medical Sciences and Conway Institute of Biomolecular and Biomedical Research, University College Dublin, Dublin 4, Ireland; 18 The University of Queensland Diamantina Institute, Translational Research Institute, Princess Alexandra Hospital, Brisbane, Queensland QLD 4102, Australia; 19 Division of Rheumatology and Fraunhofer IME-Project-Group Translational Medicine and Pharmacology, Goethe University, Frankfurt 60590, Germany

## Abstract

Psoriatic arthritis (PsA) is a chronic inflammatory arthritis associated with psoriasis and, despite the larger estimated heritability for PsA, the majority of genetic susceptibility loci identified to date are shared with psoriasis. Here, we present results from a case–control association study on 1,962 PsA patients and 8,923 controls using the Immunochip genotyping array. We identify eight loci passing genome-wide significance, secondary independent effects at three loci and a distinct PsA-specific variant at the *IL23R* locus. We report two novel loci and evidence of a novel PsA-specific association at chromosome 5q31. Imputation of classical HLA alleles, amino acids and SNPs across the MHC region highlights three independent associations to class I genes. Finally, we find an enrichment of associated variants to markers of open chromatin in CD8^+^ memory primary T cells. This study identifies key insights into the genetics of PsA that could begin to explain fundamental differences between psoriasis and PsA.

## Abstract (web summary)

Psoriatic arthritis (PsA) is a chronic inflammatory arthritis with a significant genetic component. Here, the authors analyse immune-related genetic markers in 1,962 PsA patients and 8,923 controls to identify novel PsA risk loci and highlight distinct genetic differences between psoriasis and PsA.

## Graphical abstract

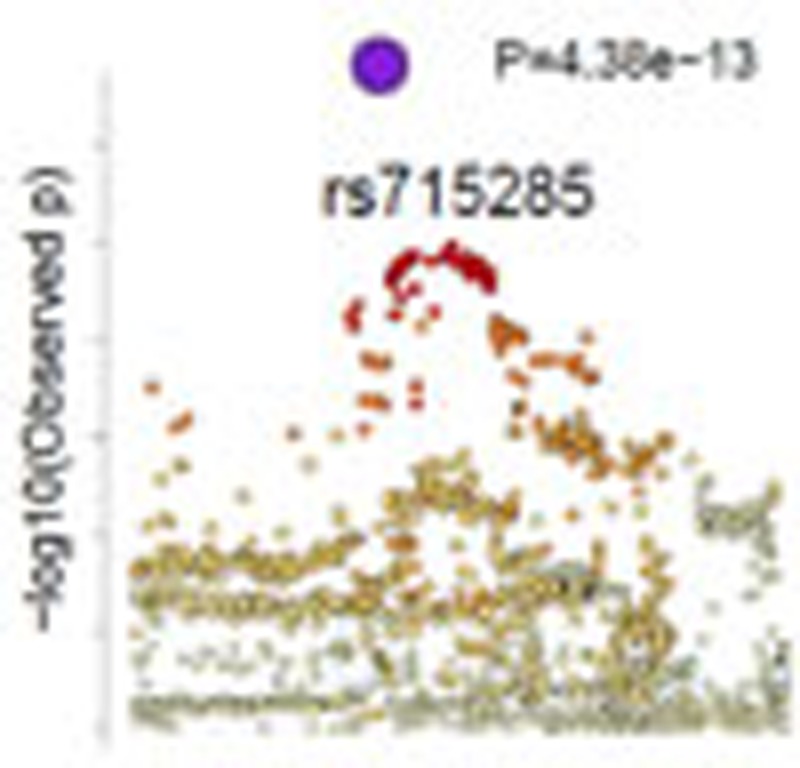

The increased prevalence of chronic inflammatory arthritis among patients with psoriasis is well described and the distinct clinical entity, referred to as psoriatic arthritis (PsA) [OMIM 607507], is now clearly recognized^1^. Prevalence rates of PsA have been estimated to be between 0.3 and 1% (ref. 2); in a recent study, 14% of a UK cohort of psoriasis patients also had co-existing PsA^3^. PsA is characterized by inflammation of the distal interphalangeal joints, sacroiliac joints and entheses; it is typically seronegative for autoantibodies and is classed as a spondyloarthritis. Its presence leads to increased morbidity and a lower quality of life than psoriasis alone^4^. The burden of illness and the socioeconomic impact of PsA has been shown to be comparable to patients with RA and ankylosing spondylitis and is estimated to result in a loss to the exchequer of over £3.5 billion per annum^5^,^6^,^7^.

Familial aggregation studies have demonstrated a strong genetic component for both psoriasis and PsA. An elegant genealogical study conducted in the Icelandic population calculated the recurrence risk ratio (*λ*_1_) for first degree relatives to be 40 for PsA^8^, while family studies for psoriasis estimate *λ*_1_ at ~8 (ref. 9). This suggests a substantial difference in the genetic architecture of the two diseases with a heavier genetic burden for PsA.

The majority of susceptibility loci identified to date are shared between the two phenotypes, which is expected, and is mediated by the presence of psoriasis in both traits. The large degree of overlap between the reported susceptibility loci indicates pleiotropic effects within shared molecular pathways for the shared skin pathology. However, given the estimated greater genetic burden for PsA, we hypothesize that PsA-specific risk loci exist. As more data on PsA genetics are published, there is an emerging evidence to support this hypothesis.

A well-established example to support genetic differentiation involves the associations to genes in the human leukocyte antigen (HLA) class I region of the major histocompatibility complex (MHC) on chromosome 6. Studies have demonstrated that certain alleles of HLA-B confer risk specifically for PsA (B*08, B*27,B*38), while HLA-C*06 is specific for psoriasis^10^. A functional polymorphism within the *MICA* gene (rs1051792) has also been suggested to be specific for purely cutaneous manifestations of psoriasis^11^. However, given the highly correlated nature of the two phenotypes and the extensive linkage disequilibrium (LD) across this region, it is difficult to confirm any disease-specific associations within the MHC.

Outside of the MHC, a number of reports have suggested distinct variants or differences in effect sizes and allele frequencies between the two traits at a number of loci including *IL23R, TRAF3IP2, FBXL19* and *REL*^12^,^13^,^14^,^15^. A number of studies have reported evidence to support the association to the *IL13* gene at chromosome 5q31 as being specific to PsA^16^,^17^,^18^. However subsequent large psoriasis studies, including subtype analysis, have all reported robust association to *IL13* (refs 19, 20). It is worth noting that some of those studies will be confounded by phenotype misclassification due to the presence of unidentified PsA patients within the psoriasis study group. To date, conclusive evidence for PsA-specific genetic risk factors outside of the HLA region has yet to emerge.

In this study, we use the Immunochip genotyping array to fine-map previously reported immune-related susceptibility loci, including known psoriasis susceptibility loci, to identify novel PsA susceptibility loci in a collection of samples from 1,962 PsA patients and 8,923 healthy population controls of Caucasian ancestry. The study reveals important insights into the genetics of PsA susceptibility as we find evidence for a distinct PsA variant at the known psoriasis susceptibility locus, *IL23R*, and we identify a new PsA-specific association at chromosome 5q31. Together, these results begin to highlight the important differences in susceptibility to PsA and psoriasis.

## Results

### Statistical quality control

Following quality control (QC), there remained 129,874 polymorphic single-nucleotide polymorphisms (SNPs) for analysis in 1,962 case and 8,923 control samples; full details of sample and SNP exclusions are described in Supplementary Tables 1 and 2. The genomic inflation factor (*λ*) was estimated to be 1.07 (standardized for an equivalent study of 1,000 cases and 1,000 controls, *λ*_1000_, the estimate was 1.02), indicating minimal population stratification (quantile–quantile plots are presented in Supplementary Fig. 1).

### Primary association analysis

The PCA-corrected analysis of all study samples showed association at genome-wide significance (*P*<5 × 10^−8^) at eight loci (Table 1, Supplementary Fig. 2). Seven of these have previously been reported for psoriasis (MHC, *TRAF3IP2*, *IL12B*, *IL23R*, *IL23A-STAT2*, *TNIP1*, *TYK2)* (Table 1). On comparison with the recently reported psoriasis Immunochip study, we found at least nominal evidence of association to the 36 previously reported loci (*P*<0.05; Table 1) suggesting substantial overlap between the two phenotypes^20^. In addition, the effect estimate was similar at loci where the SNP, or a highly correlated proxy, was associated with both the phenotypes (Supplementary Fig. 3).

### Distinct PsA risk variants at the IL23R locus

We noted that the PsA study index SNP is not always highly correlated with the reported psoriasis index SNP; notable examples include *IL23R*, *TYK2* and *NOS2A* loci (*r*^2^*<*0.2). This was investigated further by re-analysing the previously reported psoriasis risk loci in PsA while including the psoriasis index SNP as a covariate (Supplementary Table 3). Six loci remained significant (*P*<1 × 10^−4^) following conditional analysis and were further investigated by comparing model fit using likelihood ratio tests (LRT). Only one locus, *IL23R*, demonstrated true independence of the psoriasis SNP; the PsA-associated SNP rs12044149 remained highly significant (*P=*2.4 × 10^−14^) after conditioning on the psoriasis SNP, rs9988642, and the addition of rs9988642 did not improve the model fit with LRT (*P*=0.21). Although only a single effect at the *IL23R* locus has been reported for psoriasis, multiple independent risk haplotypes have been reported for ankylosing spondylitis (AS) tagged by the SNPs rs11209026, which is highly correlated with rs9988642, and rs11209032 (ref. 21). The PsA index SNP was found to be independent of the AS second effect and remains highly significant after including rs11209032 as a covariate (*P*=1.48 × 10^−15^). The data provide compelling evidence for a distinct PsA risk variant at *IL23R.*

### 5q31 is a susceptibility locus specifically for PsA

**Figure 1:**
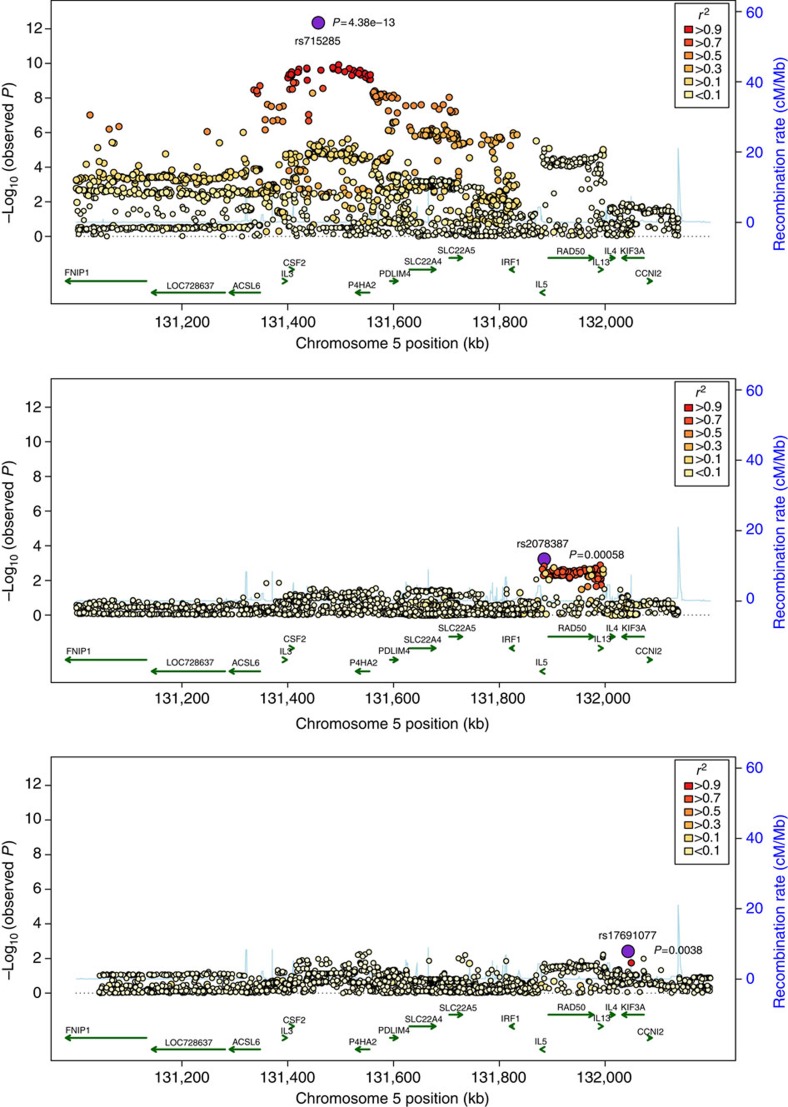
Regional association plots for chromosome 5q31 for PsA (cases=1,962, controls=8,923) and psoriasis (cases=1,784, controls=5,175). Circles represent −log_10_(*P* value) from logistic regression for imputed SNPs, colour of the circle represents linkage disequilibrium (*r*^2^) with the index SNP (purple circle). Top panel, imputed Immunochip data; middle panel, imputed Immunochip data conditioned on the index SNP rs715285; bottom panel, imputed WTCCC2 GWAS data for psoriasis, excluding known PsA samples.

We selected index SNPs from two novel loci at chromosome 5q31 and 1q31 and genotyped them in an independent cohort of PsA (*n*=864), psoriasis (*n*=1,054) and control (*n*=925) samples. In addition, genotype data were available from the WTCCC psoriasis cohort consisting of 1,784 psoriasis samples following the exclusion known PsA samples and 5,175 control samples^19^. The index SNP at chromosome 5q31, rs715285, maps to an intergenic region flanked by the genes *CSF2* and *P4HA2* and this region has been reported to be a susceptibility locus for multiple immune-related diseases including juvenile idiopathic arthritis, inflammatory bowel disease (IBD) and asthma. This association was replicated in the independent cohort of patients with PsA, and meta-analysis of PsA cohorts provides convincing evidence of association with susceptibility to PsA (*P*_validation_=4.0 × 10^−4^, *P*_meta_*=*4.38 × 10^−13^), and is independent of the previously reported associations to *IL13* (ref. 20; Table 2, Fig. 1). Interestingly, the SNP reached only nominal significance in two independent psoriasis cohorts (*P*_Erlangen_=0.05, *P*_WTCCC2_=0.21, *P*_meta_*=*0.04). Using multinomial logistic regression, we found that the effect estimates for rs715285 in PsA and psoriasis (odds ratio (OR)=1.25 and 0.99, respectively) are significantly different (*P*=7.05 × 10^−7^) providing support that this is a PsA-specific risk locus (Table 2).

Each PsA-associated region (index SNP<1.0 × 10^−4^) with sufficient marker density (minimum 100 SNPs) was imputed and we applied a Bayesian refinement approach to select credible SNP sets that best explain the observed association (Supplementary Table 4; ref. 22). In order to further prioritize SNPs with potential functional effects, the sets were functionally annotated against gene transcripts and ENCODE features. Consistent with other reports for complex traits, we find that most SNPs map to intronic and intergenic regions with large proportions mapping to elements predictive of transcriptional regulation (Supplementary Table 5). Focusing specifically on the putative PsA-specific locus at chromosome 5q31, we observe that 99% of the posterior probability for the association rests on a credible SNP set consisting of 35 SNPs from a total of 1,644 following imputation. The credible SNP set defines a 137 kb region (chr5:131,418,948–131,556,203) partially spanning the *P4HA2* gene (Supplementary Fig. 4). The functional annotation shows that most SNPS are intronic within the *P4HA2* gene or intergenic mapping to ENCODE features indicative of transcriptional activity, such as DNase hypersensitivity sites and histone modification marks (H3K4Me1). Four SNPs map to multiple ENCODE features (rs10065787, rs3846728, rs27437 and rs7721882), but of particular interest is the SNP, rs10065787, which maps to a site of multiple clusters for occupancy of transcription factors known to be important for CD8^+^ T-cell differentiation including, RUNX3, BATF and IKZF1 (refs 23, 24).

**Figure 2:**
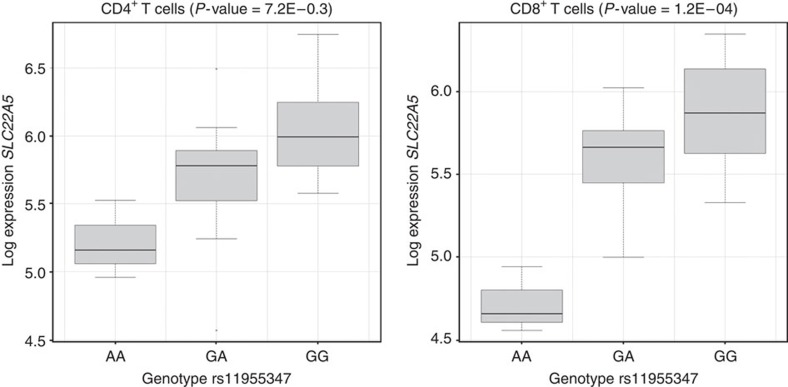
eQTL for *SLC22A5* in CD4^+^ (left) and CD8^+^ T cells (right). Log gene expression of *SLC22A5* grouped by genotype of rs11955347 (*n*=22). Linkage disequilibrium of this SNP with the index SNP, rs715285, is *r*^2^=0.7. *P*-value from linear regression of gene expression and genotype. The risk allele of rs715285 corresponds with decreased expression of *SLC22A5*.

To help prioritize candidate genes in this gene-rich region, we performed cell-specific expression quantitative trait loci (eQTL) analysis in primary cells for CD8^+^ and CD4^+^ T cells from healthy individuals. The most significant correlation between gene expression and genotype was with rs11955347 and probes in the *SLC22A5* in CD8^+^ T cells and CD4^+^ T cells (Fig. 2). This SNP is highly correlated (*r*^2^=0.7) with the index SNP, rs715285 (Fig. 2), where the risk allele, G, of rs715285 is in phase with the A allele of rs11955347, which in turn corresponds with decreased expression of *SLC22A5.*

### 1q31 is a novel psoriasis susceptibility locus

The index SNP at chromosome 1q31 (rs2477077) is located within intron 3 of the gene *DENND1B* (transcript variants 2 and 3). This gene has previously been reported to be associated with susceptibility to IBD and primary biliary cirrhosis (PBC). Our reported index SNP is highly correlated with both variants (rs2488389, *r*^2^*>*0.99 and rs2488393, *r*^2^*=*0.94, respectively)^25^,^26^. Validation of this SNP in an independent cohort of patients with PsA did not reach statistical significance (*P*=0.07). However, there was evidence to support association to this SNP in both psoriasis data sets, further substantiated by meta-analysis (*P*_Erlangen_=6.07 × 10^−3^, *P*_WTCCC2_=2.24 × 10^−5^, *P*_meta_=2.40 × 10^−7^; Table 2). Meta-analysis of all psoriasis and PsA data from Immunochip and combined validation data sets exceeded genome-wide significance (*P=*3.05 × 10^−8^; Supplementary Fig. 5). The results suggest that *DENND1B* is a susceptibility locus for psoriasis *per se*.

### Independent effects at three non-HLA loci

To identify independent secondary effects at the PsA loci, forward stepwise logistic regression was performed where the index SNP was included as a covariate. We found evidence (*P*<10^−4^) of secondary effects at three loci; *IFIH1*, *IL12B* and *NOS2* (Supplementary Table 6).

### CD8^+^ memory primary cells are critical for PsA

We next attempted to identify the relevant cell types for PsA by testing the overlap of associated variants with markers of transcriptional activity. Here, we tested 20 associated SNPs (*P*<1.0 × 10^−4^ for previously associated loci and validated novel loci) for overlap with trimethylation of histone H3 at lysine 4 (H3K4me3) peaks in 34 cell and tissues types. This method has previously been demonstrated to be effective at identifying relevant cell types for multiple complex traits^27^. The most significant association was to CD8^+^ memory primary cells (*P*=1.6 × 10^−3^) with four SNPs showing a specificity greater than the permuted 95th percentile (0.26) (Supplementary Table 7). It is interesting to note that the SNP (rs10065787) with the highest specificity for CD8^+^ memory primary cells is one of the four SNPs prioritized by functional annotation from the PsA-specific locus at chromosome 5q31 (SNP score=0.86) (Supplementary Table 8).

### Independent associations to three HLA genes

**Figure 3:**
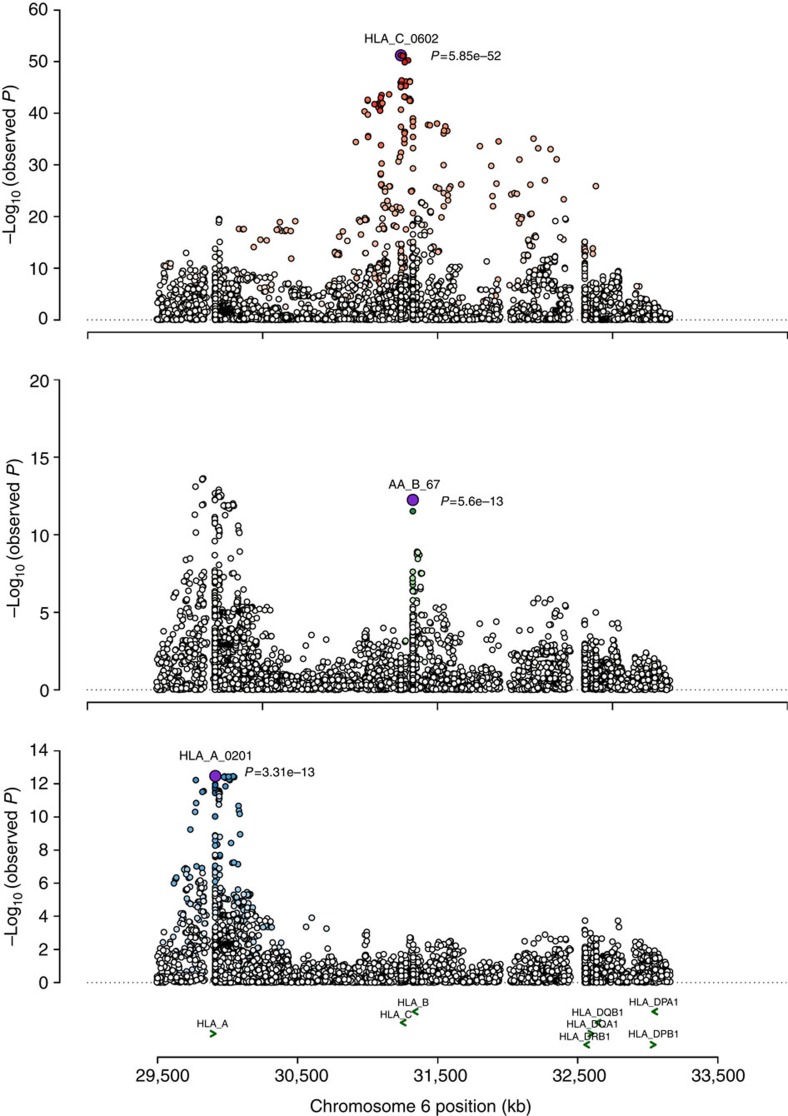
Association plots for three independent associations to HLA Class I genes. Analysis was performed in 1,464 cases and 8,469 controls from the UK. Circles represent the −log_10_(*P* value) from omnibus test for SNPs, amino acids and classical HLA alleles. *x* axis represents chromosomal base position in kilobases. Top panel, HLA-C*0602; middle panel, amino acid position 67 in HLA-B; bottom panel, HLA-A*0201.

The high quality imputed variants for the HLA region consisted of 70 classical *HLA* alleles at two-digit resolution, 88 *HLA* alleles at four-digit resolution, 335 amino acid positions and 6,830 SNPs for the subset of UK samples (cases=1,464, controls=8,469). The strongest association from the initial analysis of all imputed markers was to the classical four-digit allele of HLA-C*0602 (*P=*5.85 × 10^−52^, OR=2.36; Table 3, Fig. 3). After adjusting for the effect of HLA-C*0602, no secondary effects were found that were independent of *HLA-B.* We conclude that there are no secondary effects at *HLA-C* after adjusting for HLA-C*0602 in this data set and the second major effect is to *HLA-B*. We next conditioned on all *HLA-C* two- and four-digit alleles and found the strongest association to an amino acid at position 67 within HLA-B (*P=*1.89 × 10^−12^) where the presence of a cysteine conferred the greatest increased risk (OR=2.85; Fig. 3, Table 3 and Supplementary Fig. 6) and defines the classical HLA-B*27 allele. This is consistent with findings reported in a previous study by Okada *et al*.^28^

Proceeding with a forward stepwise logistic regression to identify markers outside *HLA-C* and *HLA-B*, by including all HLA-C and HLA-B two- and four-digit alleles in the model, we find the next most statistically significant variant was to the four-digit allele HLA-A*0201(*P*=3.31 × 10^−13^; Fig. 3). The effect at *HLA*-A*0201 could not be differentiated from that of four highly associated amino acids at positions 62, 74, 95 and 107 (Supplementary Fig. 6). No further variants reached the predefined level of significance following adjustment for *HLA-C*, *HLA-B* and HLA-A*0201.

We validated this association profile in an independent data set of 572 PsA cases and 888 controls from Germany and replicated the associations to HLA-C*0602 (*P*=1.59 × 10^−14^) and the amino acid at position 67 of HLA-B (*P=*1.89 × 10^−12^; Supplementary Table 9 and Supplementary Fig. 7; ref. 13) The association to *HLA-A* was also replicated with the presence of glycine at position 107 conferring risk (*P=*3.3 × 10^−9^) (Supplementary Fig. 7). This residue defines the classical allele four-digit allele HLA-A*0201 identified in the UK analysis and the two variants are highly correlated (*r*^2^=0.94).

## Discussion

This study demonstrates substantial allele sharing at previously reported psoriasis susceptibility loci as would be expected given the shared clinical characteristics. In addition, the study reveals key insights into the genetics of PsA that begin to explain fundamental differences between psoriasis and PsA. We identify a PsA-specific risk locus at chromosome 5q31, distinct risk variants for PsA at a known psoriasis susceptibility locus and provide a further line of evidence to support CD8^+^ T cells as a relevant cell type for pursuing functional experiments. In addition, we identify a novel risk locus for susceptibility to psoriasis *per se* at chromosome 1q31 to a variant in *DENND1B*. Finally, we report results for the imputation of HLA classical alleles and amino acids identifying three independent effects to MHC class I molecules. The results provide compelling evidence for the existence of PsA-specific risk loci that we would expect to find given the increased genetic burden estimated from family studies.

The PsA-specific association on chromosome 5q31 forms part of a large region containing many functionally interesting candidate genes. There is extensive LD across this region and it is challenging to statistically differentiate a causal SNP. However, with the integration of eQTL and ENCODE data, we were able to prioritize a single candidate gene (*SLC22A5*) and candidate SNP (rs10065787) for further investigation. The *SLC22A5* gene encodes a transmembrane cation transporter found in a wide variety of tissues and organs. It has a functional role in ergothioneine and carnitine transport in addition to control of intestinal absorption. This last point is of particular interest in the context of gut inflammation/microbiome. Given the overlap of Crohn’s disease with psoriasis and PsA, there is growing interest in the possibility that PsA may be triggered by gut microbes in genetically susceptible individuals. *SLC22A5* has previously been reported to be associated with IBD^25^, whereas previous psoriasis reports relating to chromosome 5q31 have focused on variants mapping to the *IL13* gene. The index SNP in this PsA study is statistically independent of the *IL13* psoriasis-associated variant. It should be noted that while the eQTL analysis and ENCODE annotation presented in this study are informative with regards to prioritizing SNPs and candidate genes for functional validation in the laboratory, the current data sets may not represent the true biological context. For example, eQTLs in immune cells are known to be influenced by stimulation with many eQTLs only observed under stimulated conditions^29^.

Distinct PsA SNPs have previously been reported at known psoriasis susceptibility loci^12^; here we confirm that the PsA risk variant at the *IL23R* locus is independent from the reported psoriasis variant (*P*_cond_*=*2.4 × 10^−14^) and including the reported psoriasis SNP does not improve the fit of the model (*P*_LRT_=0.21). However, we do not find evidence to support a distinct variant at the *TNFAIP3* locus as reported by Nair *et al*., as our most associated SNP variant is highly correlated with reported psoriasis Immunochip variant (*r*^2^=0.78).

Identifying disease-specific genetic risk factors has important implications for epidemiology and pharmacogenetics as it may allow the stratification of patients with psoriasis to identify those at high risk of developing PsA and potentially identify new therapeutic targets. Indeed some therapies used in the treatment of psoriasis, such as Ciclosporin and Fumaderm, do not work in the treatment of PsA; whereas others such as Sulfasalazine and Leflunomide, effective for PsA, have no or limited effect in psoriasis. There is also data to show that biological agents including antitumour necrosis factor α and Interleukins 12/23 inhibitors are effective in the treatment of both psoriasis and PsA; yet, not all patients respond predictably, an effect potentially driven in part by differing genetic factors between the two conditions.

Although the results presented here provide evidence for the existence of PsA-specific risk loci, the study design is not ideal for the detection of such loci. Future study design would involve a cross-phenotype analysis comprising large numbers of PsA cases, cutaneous only psoriasis (PsC) cases and healthy controls. This would allow a direct comparison of effects between the two phenotypes to assess pleiotropy. Power is a key issue, as in all genetic studies, and phenotype misclassification may influence power. The presence of unidentified PsA in the PsC comparison group will reduce power requiring robust phenotyping of the PsC cohort, for example, by restricting the analysis to psoriasis patients with 10 years of disease without a diagnosis of PsA. We have attempted to address the problem in the current study by excluding known PsA samples from the WTCCC2 collection on the basis of the information from contributors of the samples. In addition, the Erlangen psoriasis cohort specifically recruits patients with a disease duration of 10 years or more without a diagnosis of PsA. A number of previous psoriasis GWAS have directly compared genotypes between PsC and PsA^14^,^30^; for example, Nair *et al*. found modestly significant frequency differences between PsC and PsA for SNPs at the *HLA-C* (*P=*0.006) and *IL12B* (*P=*0.01) loci^31^. However, these studies have focused only on confirming the association of known and newly discovered psoriasis susceptibility loci in PsA as opposed to discovering novel PsA-specific loci.

Here we provide empirical evidence that PsA genetic risk variants co-localize with epigenetic markers of open chromatin preferentially in CD8^+^ memory cells. Thus, we add a further line of evidence to support their importance in the underlying disease mechanism and as a relevant cell type for pursuing functional studies. CD8^+^ T cells have been postulated as having a key role in the development of PsA based on several lines of evidence^32^. First, immunohistochemistry and flow cytometry experiments have found that CD8^+^ T cells predominate in the synovial fluid of patients with PsA compared with peripheral blood^33^. In addition, levels of these IL-17 producing CD8^+^ T cells correlate with clinical measures of disease activity and are increased in patients with erosive disease^34^. Second, the importance of CD8^+^ cells is underlined by genetic evidence; the strongest genetic associations with susceptibility to PsA are to alleles of HLA genes in the MHC class I region. Molecules encoded by these genes are responsible for the presentation of peptides to the T-cell receptor of CD8^+^ lineage T cells^10^,^35^. Outside the MHC region, associations to key genes involved in the differentiation of CD8^+^ cells, such as *RUNX3*, have been found to PsA^36^. The susceptibility variant at this locus is highly correlated with the reported AS SNP (rs6600247, *r*^2^*=*0.97) and variants at this locus have been shown to be associated with CD8^+^ T-cell counts^21^. Only four of the 20 SNPs included in our analysis were found to have high scores for CD8^+^ memory T cells, potentially highlighting the sensitivity of this method with low numbers of markers. Conclusions regarding critical cell types will become more robust as more loci are discovered.

The differentiation of CD8^+^ T cells is regulated by a number of transcription factors including BATF and RUNX3, which were identified during the bioinformatic analysis of 5q31 as a potential mechanism for the observed eQTL. It is also interesting to note that the 5q31 index SNP had the strongest individual SNP specificity score for CD8^+^ memory T cells. BATF4 has been shown to epigenetically regulate the differentiation of CD8^+^ cells via histone acetylation^37^. While in this study we focused on H3K4me3, a marker of active promoters, the chromatin marks often co-localize and there is, indeed, a H3K27Ac peak in close proximity to the index SNP. Further investigation will require genome-wide binding profiles of these transcription factors in CD8^+^ memory T cells, which in turn may refine candidate loci.

The *DENND1B* locus has previously been associated with IBD (rs2488389), PBC (rs2488393) and childhood asthma (rs2786098; refs 25, 26, 38). Our reported SNP, rs2477077, is highly correlated with both the IBD and PBC reported variants (*r*^2^>0.94) with directionally consistent effect estimates. However, very little correlation is observed with the asthma reported SNP (*r*^2^<0.08). This implies a pleiotropic locus containing both shared and distinct causal variants for different phenotypes^39^ where the psoriasis, IBD and PBC SNPs likely tag the same causal variant, but with an independent causal variant for asthma. The function of *DENND1B* is not fully understood, but it has been found to be upregulated in T cells providing support for the relevance of this type of cell in PsA and psoriasis aetiology. The data from our study suggest that this is a susceptibility locus for psoriasis irrespective of the presence of inflammatory arthritis.

We identified and validated three independent effects to MHC class I genes, with main effects to the imputed four-digit allele HLA-C*0602, amino acid at position 67 of HLA-B and HLA-A*0201. These associations to the three different genes were replicated in an independent data set; however, the differing primary associations at the *HLA-A* locus between the discovery and validation cohorts highlight the challenges in identifying the true causal variant in a region of high LD. The variants identified at *HLA-A* for both data sets are highly correlated and considered to tag the same effect. The associations to these loci have previously been reported for psoriasis and, consistent with previous studies, we find the effect estimate at HLA-C*0602 to be lower for PsA than that reported for psoriasis, with OR of 2.34 and 3.26, respectively^28^. The effect estimate and direction at *HLA-B* and *HLA-A* are similar to that reported for psoriasis. Associations to HLA-B*27 and HLA-A*0201 have previously been reported to AS^21^, providing further support of genetic overlap between these two conditions. The direction of effect in PsA at these loci is the same as AS, but the magnitude of effect at HLA-B*27 is substantially lower for PsA (OR=46 and OR=1.90, respectively).

Associations to *HLA-B* have previously been reported to anti-citrullinated-protein-autoantibody-negative (ACPA^−^) rheumatoid arthritis (RA) where an aspartate at position 9 has been reported to increase the risk^40^. However, no associations were observed to this amino acid (*P=*0.88) or the related HLA-B*08 allele (*P=*0.49) in our PsA samples. Furthermore, no convincing evidence for association was observed for the other reported ACPA^−^ RA association to HLA_DRB1*03 (*P=*0.36, amino acid position 11; *P=*0.02).

PsA is a clinically heterogeneous disease and it has been shown that certain clinical sub-phenotypes correlate with particular HLA alleles, for example HLA-B*27 has been shown to be associated with the extent and severity of axial involvement^41^. Future studies would greatly benefit from the incorporation of clinical information to help resolve the true nature of associations across this region^39^. This heterogeneity will also confound the naive comparison of results across cohorts where the relative proportions of clinical subtypes may differ.

In summary, this study identifies key insights into the genetics of PsA that begin to explain fundamental differences between psoriasis and PsA. We identify a PsA-specific risk locus at chromosome 5q31 where functional annotation and cell-specific gene expression data identify putative candidate SNP (rs100657871) and gene (*SLC22A5)* that can be prioritized for functional evaluation. In addition, the study demonstrates the existence of distinct risk variants at a known susceptibility locus (*IL23R*) and provides a further line of evidence to support CD8^+^ T cells as a relevant cell type for pursuing functional experiments. We identify a novel risk locus for susceptibility to psoriasis *per se* at chromosome 1q31 to variant in *DENND1B*. Finally, we report results for the imputation of HLA classical alleles and amino acids identifying three independent effects to MHC class I molecules. The results provide compelling evidence for the existence of PsA-specific risk loci, which is not unexpected, given the increased genetic burden estimated from family studies.

## Methods

### Genotyping

Samples were genotyped using Immunochip, an Illumina iSelect HD custom genotyping array, in accordance to the manufacturer’s instructions at five genotyping centres: the Wellcome Trust Sanger Institute, the Centre for Public Health Genomics at The University of Virginia, Arthritis Research UK Centre for Genetics and Genomics at The University of Manchester, the University of Queenland Diamantina Institute, and the Institute of Molecular Medicine at The University of Dublin.

### Genotype calling and quality control

All case and control idat files were collated at the Arthritis Research UK Centre for Genetics and Genomics laboratory at The University of Manchester. Genotype clustering and calling was performed using the GenomeStudio Data Analysis software platform (Genotyping Module v1.8.4) as a single project using the Illumina manifest file Immuno_BeadChip_11419691_B.bpm (NCBI Build 36, hg18). Preliminary genotype clustering was performed using the default Illumina cluster file Immunochip_Gentrain_June2010.egt to identify poor quality sample (call rate<0.90). Following exclusion of these samples, automated reclustering was performed to calibrate cluster on the basis of the study samples. An extensive manual review of genotype clusters was performed on the basis of ranking of the quality metrics; cluster separation (<0.4), signal intensity (<1.0), call rate (<0.98) and allele frequency. SNPs with a cluster separation <0.4 and a call rate <0.95 were non-polymorphic, duplicates or mapping to chromosome Y or the mitochondria were excluded before further sample QC.

### Statistical quality control

Samples were excluded with a call rate <0.98 or if they were identified as being an outlier on the basis of autosomal heterozygosity (3 s.d. from the mean autosomal heterozygosity of samples passing the call rate threshold). Duplicate and related individuals were identified using identity-by-descent, performed in PLINK using a set of 18,664 LD pruned (*r*^2^) SNPs with a minor allele frequency (MAF) >0.05. For each pair of individuals with a PI_HAT >0.2, the sample with the lowest call rate was excluded. Principal component analysis (PCA), using EIGENSOFT (v4.2) and the LD pruned SNP set, was performed to infer ancestry (Supplementary Fig. 8). Outliers of the main cluster were identified using a nearest-neighbour clustering algorithm based on the first two principal components (performed using the R package nnclust (version 2.2)). A final round of SNP filtering was performed on the remaining high quality samples; this included a call rate threshold of ≥0.98 in cases and controls, exclusion of SNPs with significant deviation from Hardy–Weinberg equilibrium <5 × 10^−7^ in controls, and a MAF <0.01.

### Association testing

Case–control association testing was performed using logistic regression including the top two principal components as covariates in PLINK (v1.07). Principal components were generated for all the samples passing QC, as described above. Cluster plots for all reported SNPs were manually checked and poor-performing SNPs were removed. At selected loci, effects independent of the index SNPs were determined by forward stepwise logistic regression including the index SNP as a covariate. Genomic inflation was calculated using a set of 2,840 SNPs passing QC that were included as part of either a reading and writing or psychosis and schizophrenia study. SNPs mapping to previously reported psoriasis susceptibility loci were excluded to create a null set of SNPs to estimate the overall inflation of test statistics using genomic control using the R package gap (version 1.1–10).

### Conditional analysis

We performed conditional analysis using LRT to determine which model represented the best fit. The aim was to test if adding additional genetic markers (alternative model), such as known psoriasis SNPS, significantly improved the models fit for phenotypic variance over the original null model. A significant LRT *P* value indicates that the alternative model explains a greater proportion of the phenotypic variation and all markers in the model are relevant to disease. The Akaike information criterion and the Bayesian information criterion were used to assess model fitness where a decrease in either is suggestive of improved fitness with minimal parameters.

In the case of previously described susceptibility loci we include the reported psoriasis SNP, or multiple SNPs where independent effects have been observed, as covariates in the regression model. We interpret a situation where the PsA SNP remains significant after conditioning on known psoriasis SNPs and where adding the known psoriasis SNP to the model does not significantly improve the fit as evidence to support a distinct variant for PsA.

### Multinomial logistic regression

To test if the effect estimates for rs715285 were significantly different between PsA and psoriasis, we performed a multinomial logistic regression (Stata version 13) including the first two principal components as covariates. The genotype data for rs715285 from WTCCC psoriasis samples (*n*=1,784 following exclusion of known PsA samples) was combined with the PsA immunochip data. The genotyping rate in the combined data set for rs715285 was >99.9%.

### Validation of novel loci

The index SNP for each novel locus passing 5.0 × 10^−6^ was selected for validation genotyping in independent cohorts of 864 PsA cases, 1,054 psoriasis cases and 925 healthy control samples by the University of Erlangen. Meta-analysis of the German and Immunochip PsA summary statistics was performed using PLINK (v1.07) using inverse variance and assuming a fixed effect across the two populations. In addition, we explored these associations in the WTCCC2 GWAS psoriasis data set, after excluding known PsA patients (*n*=394) providing a data set of 1,784 psoriasis patients and 5,175 controls^19^. Meta-analysis of the German and WTCCC2 psoriasis summary statistics was based on *P* values weighted for study sample size performed in R using the gap package. An overall analysis was performed by meta-analysis of the combined Erlangen data and Immunochip data to test for association to psoriasis *per se*. The WTCCC2 data set was not included in this analysis because of large overlap in control samples.

### Fine mapping

Selected loci were phased and imputed using the PsA Immunochip data set and, for the 5q31 locus, the WTCCC2 psoriasis data set. Phasing was performed with SHAPEIT2 (v2) and imputation was performed with Impute2 (v2.3) using the 1000 Genomes integrated variant data set version 3 as the reference panel. Post-imputation analysis was restricted to SNPs with an information score ≥0.9 in addition to SNP QC defined above and performed using SNPTEST (v2.5). For each locus, we applied a Bayesian refinement approach to define the subset of SNPs that, based on posterior probability, is 99% likely to contain the causal disease-associated SNP^22^ (https://github.com/chr1swallace/finemap-psa). These credible SNP sets were then annotated for putative function based on transcript location (refseq gene annotation) and localization to ENCODE features (H3K4Me1, H3K4Me3, H3K27Ac, DNase I hypersensitivity and transcription factor ChIP-Seq clusters) using Annovar^42^.

### PBMC purification and genotyping

Twenty-three healthy volunteers were recruited at the University of Manchester as part of the National Repository Healthy Volunteers (NRHV) study. All the samples were collected with ethical committee approval (MREC 99/8/84) and all individuals provided informed consent. The median age of the subjects was 50.5 years (range=26–82 years) with eight males and 15 females. A total of 20 ml peripheral blood was collected in Vacutainer plus tubes containing EDTA (Becton Dickinson). PBMCs were extracted within 2 h of collection from 20 ml of whole blood using Ficoll Plus density gradient centrifugation (GE Healthcare). Cells were washed twice with MACS running buffer (phosphate-buffered saline (PBS), bovine serum albumin, EDTA and sodium azide; Miltenyi) and total PBMCs counted using the CASY cell counter (Roche). To allow collection of all the samples, PBMC samples were immediately cryopreserved in 1 ml recovery cell-freezing medium (Gibco) per 5 × 10^6^ cells at a cooling rate of −1 °C per minute. Samples were genotyped using the Illumina HumanCoreExome v1.0 array, in accordance with the manufacturer’s instructions. Genotype clustering and calling was performed using the GenomeStudio Data Analysis software platform using the Illumina HumanCoreExome-24 v1.0 Manifest File.

### CD4^+^ and CD8^+^ cells separation

Automated magnetic activated cell separation methods were used to positively separate CD4^+^ and CD8^+^ T lymphocytes from thawed PBMCs, as per manufacturer’s instructions (Miltenyi). Typical yields of CD4^+^ lymphocytes obtained were between 9.5 × 10^5^ and 2.69 × 10^6^ cells; whereas typical yields of CD8^+^ lymphocytes were between 4.7 × 10^5^ and 2.7 × 10^6^ cells. Cell population purities were then assessed using a Cyan ADP flow cytometer (Becton Dickinson) with samples containing on average 95.31% CD3^+^CD4^+^ cells (range=90.21–98.78%) and 99.4% CD8^+^ cells (range=94.8–100%). All the samples were immediately suspended in 1 ml Trizol reagent (Gibco) for RNA extraction.

### RNA extraction and whole transcriptome gene expression

Total RNA was extracted from CD4^+^ and CD8^+^ lymphocytes using Trizol reagent (Gibco) and 1-Bromo-3-chloropropane (BCP) (Sigma Aldrich) in 2 ml heavy phase lock gel tubes (5 prime), according to manufacturer’s instructions. To reduce the risk of genomic DNA contamination, a DNase treatment was performed using DNase I (Invitrogen) and acid phenol chloroform (Sigma Aldrich). Sample quality control was performed using the Nanodrop N1000 (Fisher scientific) and Bioanalyzer 2100 (Agilent). RNA amplification of 400 ng total RNA was performed using the Illumina TotalPrep Amplification Kit (Ambion), according to manufacturer’s instructions. Biotin-labelled cRNAs were then hybridized to the HumanHT-12 v4 Expression (Illumina), according to manufacturer’s instructions.

### Cell-specific eQTL

GenomeStudio (Illumina) software was used to assess control probe summary statistics and summarize bead level data. Quality control and differential analysis of summary data was performed using the limma (linear models for microarray data) bioconductor package. Data were quantile normalized and subject to log2 transformation Potential batch effects were assessed by visual inspection of MDS plots and PCA analysis.

Within the 5q31 region, expression data for 18 probes and genotype data for 104 SNPs across 22 CD8^+^ and 22 CD4^+^ samples were available. Gene expression was correlated with genotype with the use of PLINK software (version 1.07), where association testing was carried out between the genotype data and the expression data for each probe represented in the region. This was performed separately for the CD8^+^ and the CD4^+^ data sets.

### HLA imputation

The snp2hla (v1.0) software package (http://www.broadinstitute.org/mpg/epigwas/) was used to impute classical HLA alleles, amino acids and SNPs within the HLA region (chr6:29-34, hg19) using a reference panel of 5,225 T1DGC samples^43^. Analysis was performed in the UK subset of samples using best-guess genotypes from variants passing the QC thresholds: information score ≥0.9, MAF ≥0.01 and significant deviation from Hardy–Weinberg equilibrium in controls >1.0 × 10^−3^.

Analysis of all markers was performed using logistic regression assuming an additive effect based on the carriage of alleles. Given that HLA region has been demonstrated to show geographic variability across the UK^44^, the logistic regression was performed with the inclusion of the first two principal components, derived from the UK study sample set, as covariates. For multi-allelic sites, such as amino acids, we identified the most common residue or allele in the control population, which was selected as the reference and excluded from the model. The *P* value for each marker was derived from an omnibus test performed with a log-likelihood ratio test of the null and fitted models. In the first instance, the null model is comprised solely of the first two principal components and the fitted model includes the marker to be tested^45^,^46^. To identify independent effects, we performed a forward stepwise logistic regression where the top marker, ranked by the log-likelihood *P* value, was included as a covariate by addition to the null model. This was repeated until no further marker reached a predefined significance threshold of 6.8 × 10^−6^, which is a Bonferroni corrected type I error rate based on the analysis of 7,323 markers. In addition, variants were only considered to be independent if they were significantly associated in all the previous iterations of the stepwise logistic regression. To account for all the effect at a particular HLA gene, we included all two- and four-digit alleles as covariates in the regression model. Effect estimates were calculated by performing a logistic regression including the first two principal components and the markers identified in the stepwise regression as covariates^28^. Validation was performed with imputation from existing GWAS data of an independent cohort of 572 cases and 888 controls from the German population^13^.

### Phenotypic cell type specificity

The epigwas software package (http://www.broadinstitute.org/mpg/epigwas/) was used to identify the most phenotypically related cell type^27^. All PsA-associated non-HLA SNPs (*P*<1.0 × 10^−4^) were used in the analysis, which involves the colocalisation of trait-associated SNPs to the epigenetic chromatin mark H3K4me3 (trimethylation of histone H3 at lysine 4) in 34 cell and tissue types. The underlying hypothesis is that variants of a particular phenotype modify gene expression regulatory elements in a cell type relevant to that phenotype. In turn, these variants should overlap chromatin marks within that cell type.

## Additional information

**How to cite this article:** Bowes, J. *et al*. Dense genotyping of immune-related susceptibility loci reveals new insights into the genetics of psoriatic arthritis. *Nat. Commun.* 6:6046 doi: 10.1038/ncomms7046 (2015).

## Supplementary Material

Supplementary Information Supplementary Figures 1-8 and Supplementary Tables 1-9

## Figures and Tables

**Table 1:** Association statistics for the 36 previously reported psoriasis susceptibility loci.

SNP	Chr.	Position (bp)	Notable genes	Risk/non-risk allele	RAF (case)	RAF (control)	*P*-value	OR	Tsoi SNP	*r*^2^ Tsoi SNP
rs33980500	6	111,913,262	* TRAF3IP2 *	T/C	0.11	0.07	2.65E−16	1.6	rs33980500	1
rs4921482	5	158,764,478	*IL12B | ADRA1B*	T/C	0.74	0.67	1.47E−15	1.4	rs4379175	0.85*
rs12044149	1	67,600,686	* IL23R *	T/G	0.32	0.25	2.25E−15	1.4	rs9988642	0.14
rs2020854	12	56,743,367	* STAT2 *	T/C	0.96	0.93	7.73E−10	1.7	rs2066819	0.99
rs34725611	19	10,477,067	* TYK2 *	A/G	0.76	0.71	2.99E−09	1.3	rs34536443	0.09
rs76956521	5	150,464,641	*TNIP1 | ANXA6*	C/A	0.08	0.05	4.98E−09	1.5	rs2233278	1
rs4795067	17	26,106,675	* NOS2A *	G/A	0.39	0.34	1.94E−07	1.2	rs28998802	0.06
rs984971	2	163,224,521	* IFIH1 *	T/G	0.68	0.64	3.62E−06	1.2	rs17716942	0.3
rs7523412	1	25,294,264	*RUNX3 | SYF2*	A/G	0.54	0.5	5.42E−06	1.2	rs7536201	0.99
rs848	5	131,996,500	* IL13 *	C/A	0.85	0.82	1.05E−05	1.2	rs1295685	0.96
rs7552167	1	24,518,643	*IL28RA | GRHL3*	G/A	0.89	0.86	1.53E−05	1.3	rs7552167	1
rs6063454	20	48,590,791	*ZNF313 | SNAI1*	G/T	0.63	0.59	2.90E−05	1.2	rs1056198	0.9
rs1306395	2	61,076,272	*PAPOLG | REL*	T/C	0.61	0.57	2.99E−05	1.2	rs62149416	0.77
rs1133071	9	32,455,674	* DDX58 *	C/T	0.34	0.3	3.36E−05	1.2	rs11795343	0.18
rs892085	19	10,818,092	* ILF3 *	T/C	0.59	0.55	4.02E−05	1.2	rs892085	1
rs2298428	22	21,982,892	* LOC150223 *	T/C	0.2	0.18	4.38E−05	1.2	rs4821124	0.96
rs6713082	2	62,516,544	*B3GNT2 | TMEM17*	A/C	0.27	0.24	4.59E−05	1.2	rs10865331	0.29
rs8016947	14	35,832,666	*PSMA6 | LOC122589*	G/T	0.6	0.56	9.65E−05	1.2	rs8016947	1
rs4796659	17	40,590,029	*PTRF | ATP6V0A1*	G/T	0.93	0.91	0.000132	1.3	rs963986	0.01
rs62376445	5	96,196,721	*ERAP1 | ERAP2*	C/A	0.96	0.94	0.000174	1.4	rs27432	0.02
rs610604	6	138,199,417	* TNFAIP3 *	G/T	0.35	0.32	0.000325	1.1	rs582757	0.78
rs7761186	6	512,951	*EXOC2 | LOC727827*	T/C	0.99	0.98	0.000709	1.8	rs9504361	0.01
rs645078	11	64,135,298	* RPS6KA4 *	A/C	0.64	0.61	0.00086	1.1	rs645078	1
rs11121129	1	826,8095	*ERRFI1 | SLC45A1*	A/G	0.33	0.3	0.000934	1.1	rs11121129	1
rs4936059	11	128,502,496	*ETS1 | FLI1*	G/A	0.35	0.32	0.001475	1.1	rs3802826	0
rs12928822	16	11,403,893	*PRM1 | C16orf75*	C/T	0.84	0.81	0.001593	1.2	rs367569	0.56
rs6693105	1	152,590,663	*LCE3B | LCE3A*	C/T	0.68	0.65	0.002892	1.1	rs6677595	1
rs7197717	16	31,083,075	* ZNF668 *	C/A	0.41	0.38	0.003544	1.1	rs12445568	0.92
rs4561177	11	109,962,432	*LOC260340 | ZC3H12C*	A/G	0.6	0.57	0.003782	1.1	rs4561177	1
rs73112675	7	37,379,030	* ELMO1 *	G/A	0.2	0.17	0.004116	1.1	rs2700987	0.18
rs602422	18	51,805,130	* POLI *	C/T	0.31	0.29	0.004767	1.1	rs545979	1
rs1972346	10	81,067,480	* ZMIZ1 *	C/G	0.61	0.59	0.008258	1.1	rs1250546	0.4
rs11652075	17	78,178,893	* CARD14 *	C/T	0.51	0.49	0.01423	1.1	rs11652075	1
rs1973919	6	159,440,727	*RSPH3 | TAGAP*	C/T	0.33	0.31	0.01812	1.1	rs2451258	0.22
rs12236285	9	110,855,243	*LOC392382*	A/G	0.08	0.07	0.03838	1.2	rs10979182	0.03

bp, base pair; Chr, chromosome; OR, odds ratio; RAF, risk allele frequency.

^*^Tsoi secondary effect.

**Table 2:** Association statistics for two novel susceptibility loci at chromosome 1q31 and 5q31.

**SNP**	**Chr.**	**Position (bp)**	**Notable genes**	**Risk/non-risk allele**	**Stage**	**Sample**	**Phenotype**	**RAF (case)**	**RAF (control)**	***P***-**value**	**OR**	**Meta-analysis** ***P***-**value**
rs2477077	1	197,671,115	* DENND1B *	T/C	Discovery	Immunochip	PsA	0.26	0.22	1.20E−6	1.23	2.36E−7
					Validation	Erlangen	PsA	0.21	0.19	0.07	1.17	
					Validation	Erlangen	Psoriasis	0.22	0.19	6.07E−3	1.25	2.40E−7
					Validation	WTCCC2	Psoriasis	0.25	0.21	2.24E−5	1.21	
rs715285	5	131,485,383	*CSF2* | *P4HA2*	G/A	Discovery	Immunochip	PsA	0.48	0.46	2.654E−10	1.25	4.38E−13
					Validation	Erlangen	PsA	0.49	0.43	4.04E−4	1.27	
					Validation	Erlangen	Psoriasis	0.46	0.43	0.05	1.14	0.04
					Validation	WTCCC2	Psoriasis	0.47	0.46	0.21	1.05	

bp, base pair; Chr, chromosome; OR, odds ratio; RAF, risk allele frequency.

**Table 3:** Three independent associations to HLA class I genes in the UK Immunochip data set.

Gene	Position	Allele/residue	Omnibus *P* value	A1/A2	*P*-value*	Minor allele freq.		Odds ratio*	CI*
						**Cases**	**Controls**		
* HLA-C *	*—*	*0602	5.85 × 10^−52^	P/A	—	0.19	0.09	2.34	2.02:2.71
* HLA-B *	67	Cysteine	5.6 × 10^−13^	P/A	6.53 × 10^−14^	0.15	0.12	1.62	1.43:1.84
		Phenylalanine		P/A	7.44 × 10^−6^	0.23	0.25	1.29	1.15:1.44
		Methionine		P/A	7.89 × 10^−5^	0.1	0.05	1.45	1.20:1.72
		Tyrosine		P/A	0.79	0.13	0.17	1.02	0.89:1.26
		Serine		P/A	ref	0.39	0.42	ref	ref
* HLA-A *	—	*0201	3.31 × 10^−13^	P/A	—	0.34	0.28	1.39	1.27:1.52

A, absent; A1, allele 1; A2, allele 2; CI, confidence interval; freq, frequency; HLA, human leukocyte antigen; P, present.

^*^Calculated in a regression including HLA-C*0602, HLA-B amino acid position 67 and HLA-A*0201.
