# Supplementary material for: Dense genotyping of immune-related susceptibility loci reveals new insights into the genetics of psoriatic arthritis
Source: Nat Commun. 2015 Feb 5;6:6046. doi: 10.1038/ncomms7046 (PMC4327416; doi:10.1038/ncomms7046)
Supplement: Supplementary Information — Supplementary Figures 1-8 and Supplementary Tables 1-9 [file ncomms7046-s1.pdf]

Supplementary information

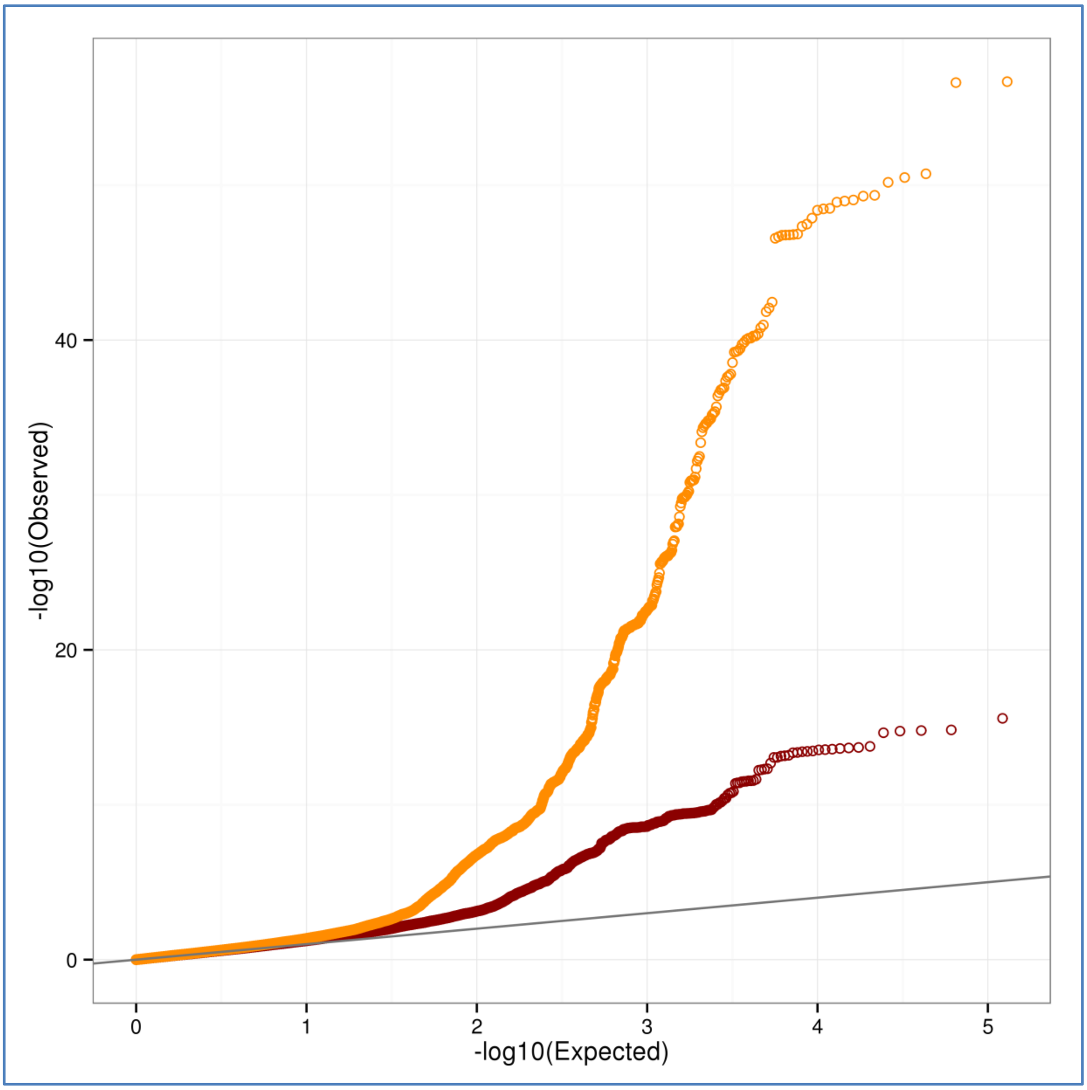

Supplementary Figure 1. Quantile-quantile (Q-Q) plot of single point association p-values from PCA corrected logistic regression for PsA Immunochip on 1,962 cases and 8,923 controls. The genomic inflation factor (λ) is 1.07 (λ_{1000} = 1.02). Orange points represent the p-value distribution from the full dataset; red points represent the p-value distribution excluding variants mapping to the HLA region.

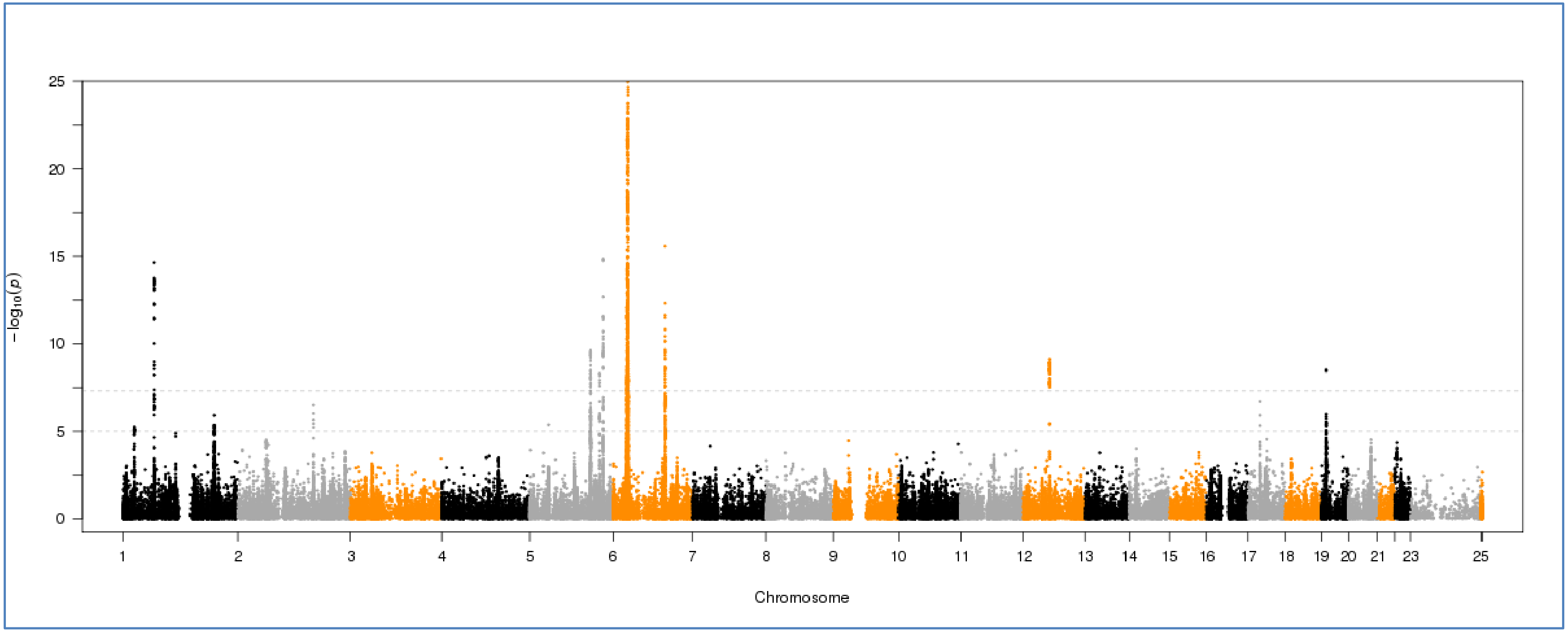

Supplementary Figure 2. Manhattan plot of PCA corrected logistic regression for PsA Immunochip on 1,962 cases and 8,923 controls**.** X-axis is chromosomal location and y-axis is –log10 of the observed p-value.

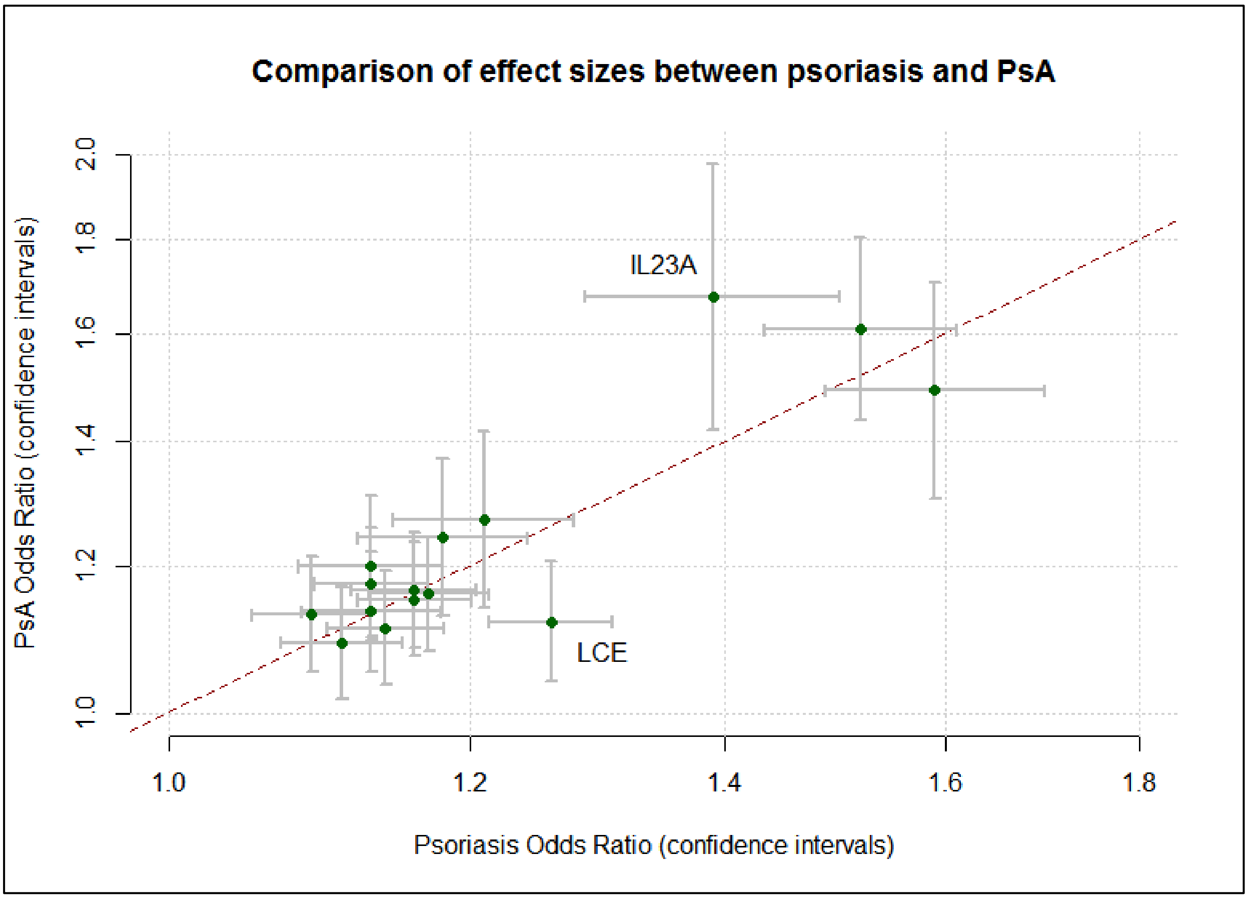

Supplementary Figure 3: Comparison of effect sizes between psoriasis and PsA**.** Effect size comparison of previously reported psoriasis susceptibility loci from Tsoi *et al (x-axiz)* and the PsA immunochip study (y-axis) where the SNP (or suitable proxy) is shared between the studies. Green dots represent the effect estimate (Odds Ratio) from each study and the grey bars indicate the 95% confidence intervals. The figure illustrates similar effect sizes at these loci with the exception of *IL23A* and the *LCE3B* loci, however confidence intervals at these two loci overlap.

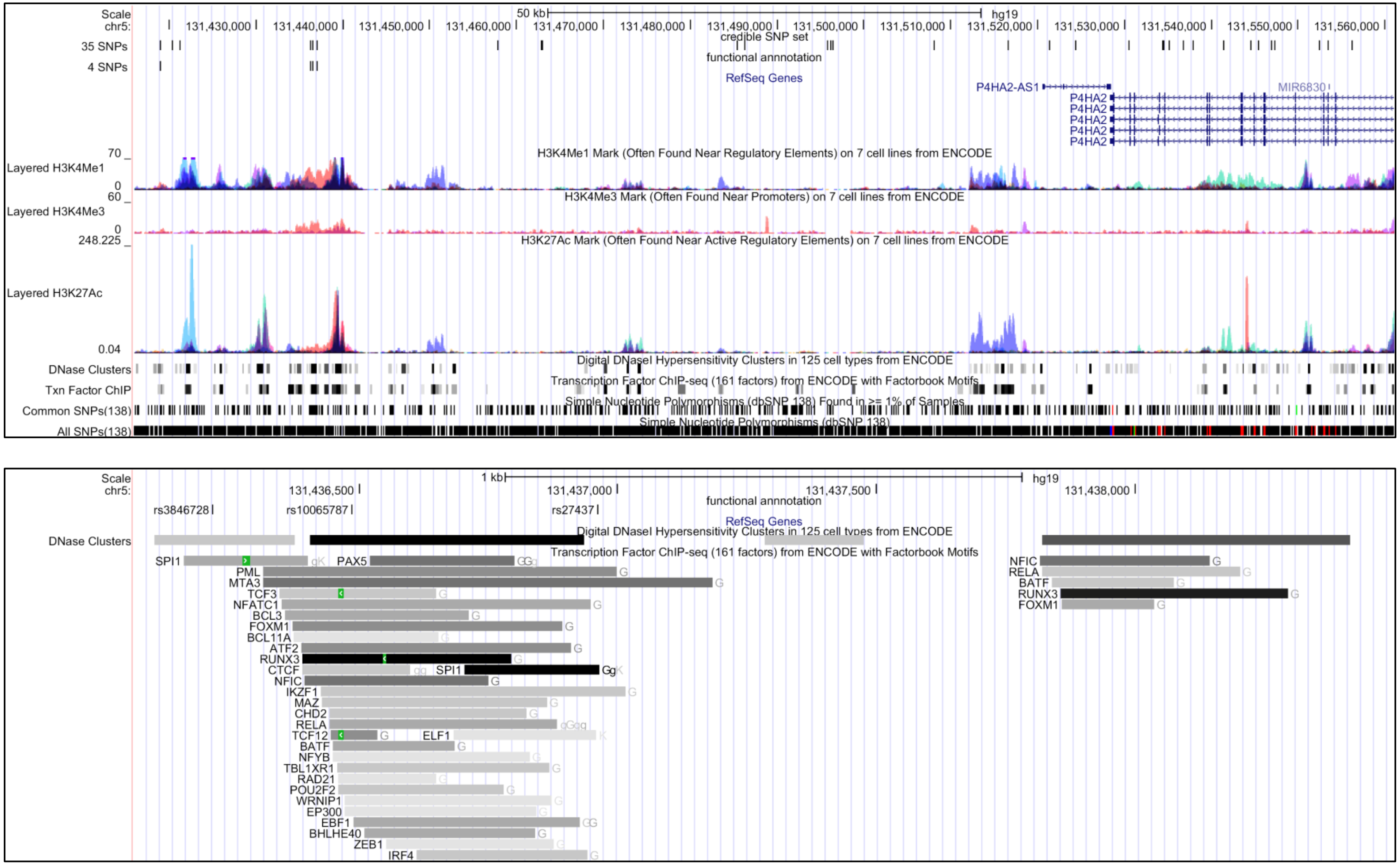

Supplementary Figure 4: Functional annotation of 5q31 credible SNP set. UCSC images showing 137kb genomic interval defined by 35 SNPs from the credible SNP set and the four SNPs prioritised by functional annotation (Top panel). SNP rs10065787 maps to multiple transcription factor sites important in CD8+ T-cell differentiation (Bottom panel).

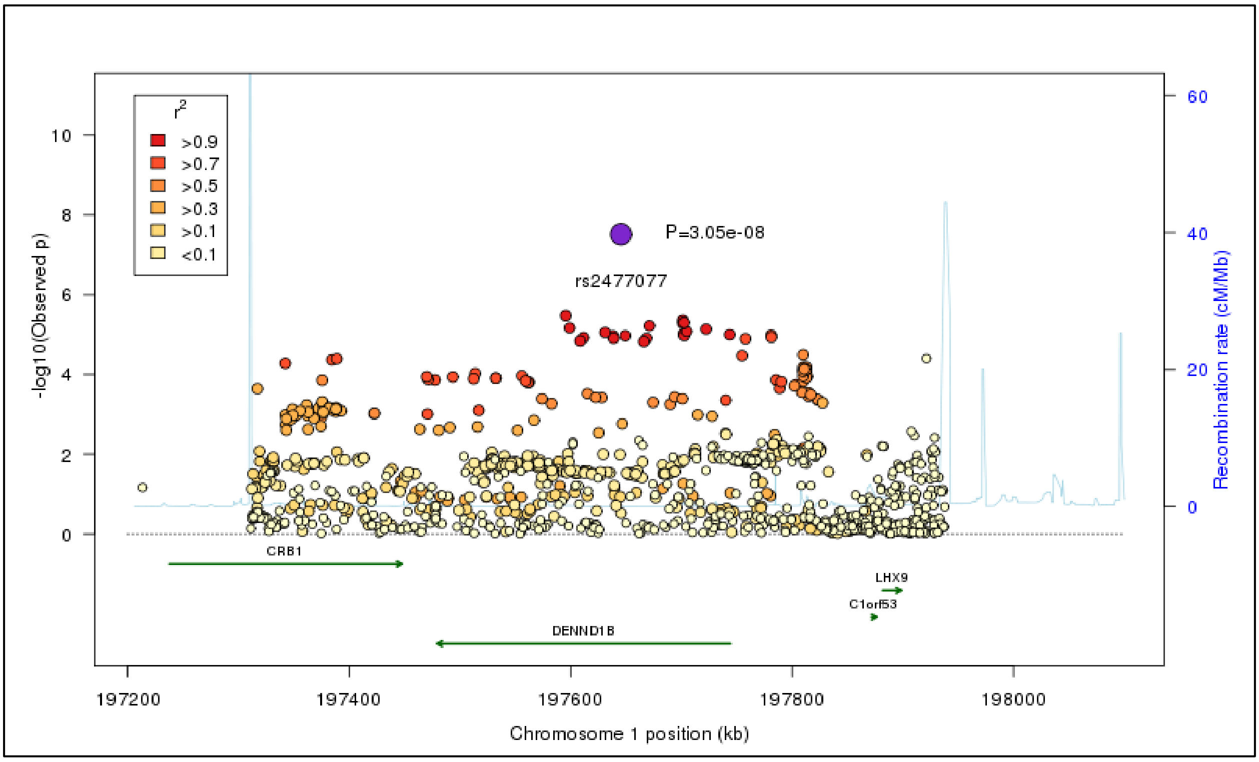

Supplementary Figure 5: Association plot of novel psoriasis susceptibility locus on chromosome 1q31 mapping to the *DENND1B* gene. X-axis is chromosomal position and gene position, y-axis -log10 of observed p-value from the logistic regression, secondary y-axis illustrates recombination rate. Circles represent p-value of SNPs and colour of filled circle reflects linkage disequilibrium (r^2) with rs2477077. Purple filled circle represents meta-analysis of discovery and validation data for rs2477077 (cases = 3880, controls = 9848)

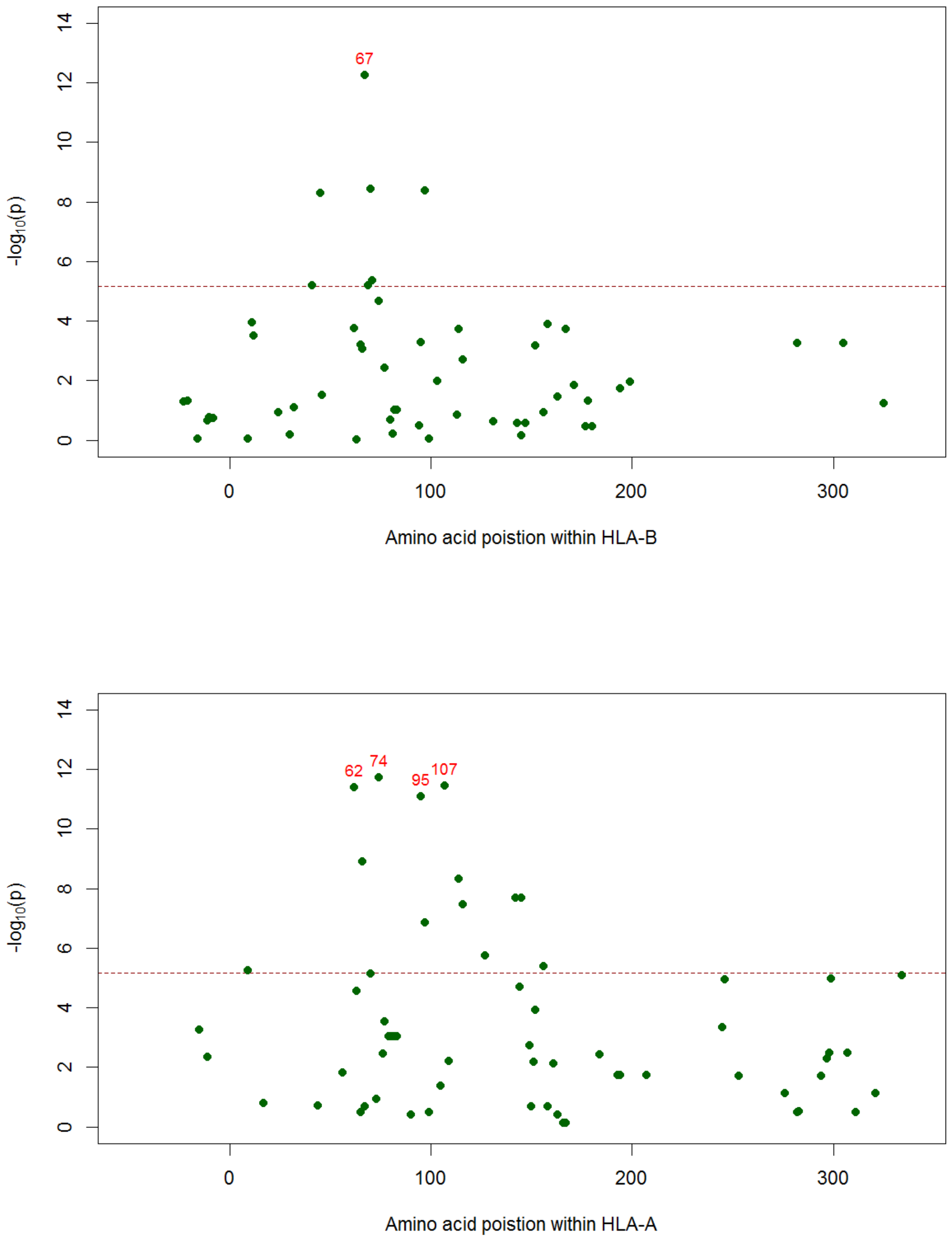

Supplementary Figure 6: Amino acid association plots for *HLA-B* and *HLA-A* in the UK Immunochip study. X-axis represents the position of the amino acid in the protein, x-axis is –log10 of the observed p-value from the omnibus test. Green circles represent individual amino acid positions and the positions of the top amino acids are labelled in red. Red dotted line indicates Bonferroni corrected significance threshold for HLA markers.

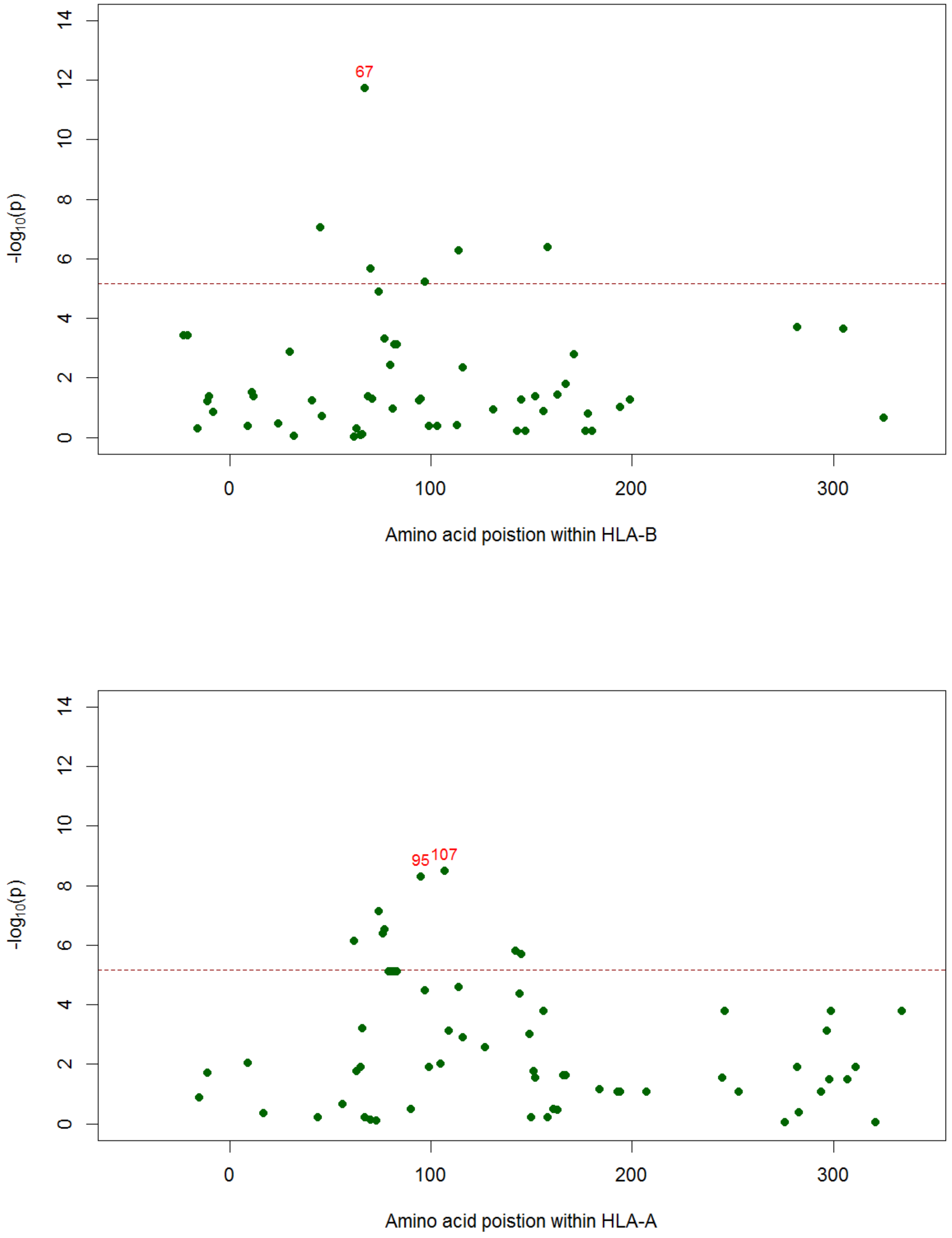

Supplementary Figure 7: Amino acid association plots for *HLA-B* and *HLA-A* in the German validation study. X-axis represents the position of the amino acid in the protein, x-axis is –log10 of the observed p-value from the omnibus test. Green circles represent individual amino acid positions and the positions of the top amino acids are labelled in red. Red dotted line indicates Bonferroni corrected significance threshold for HLA markers.

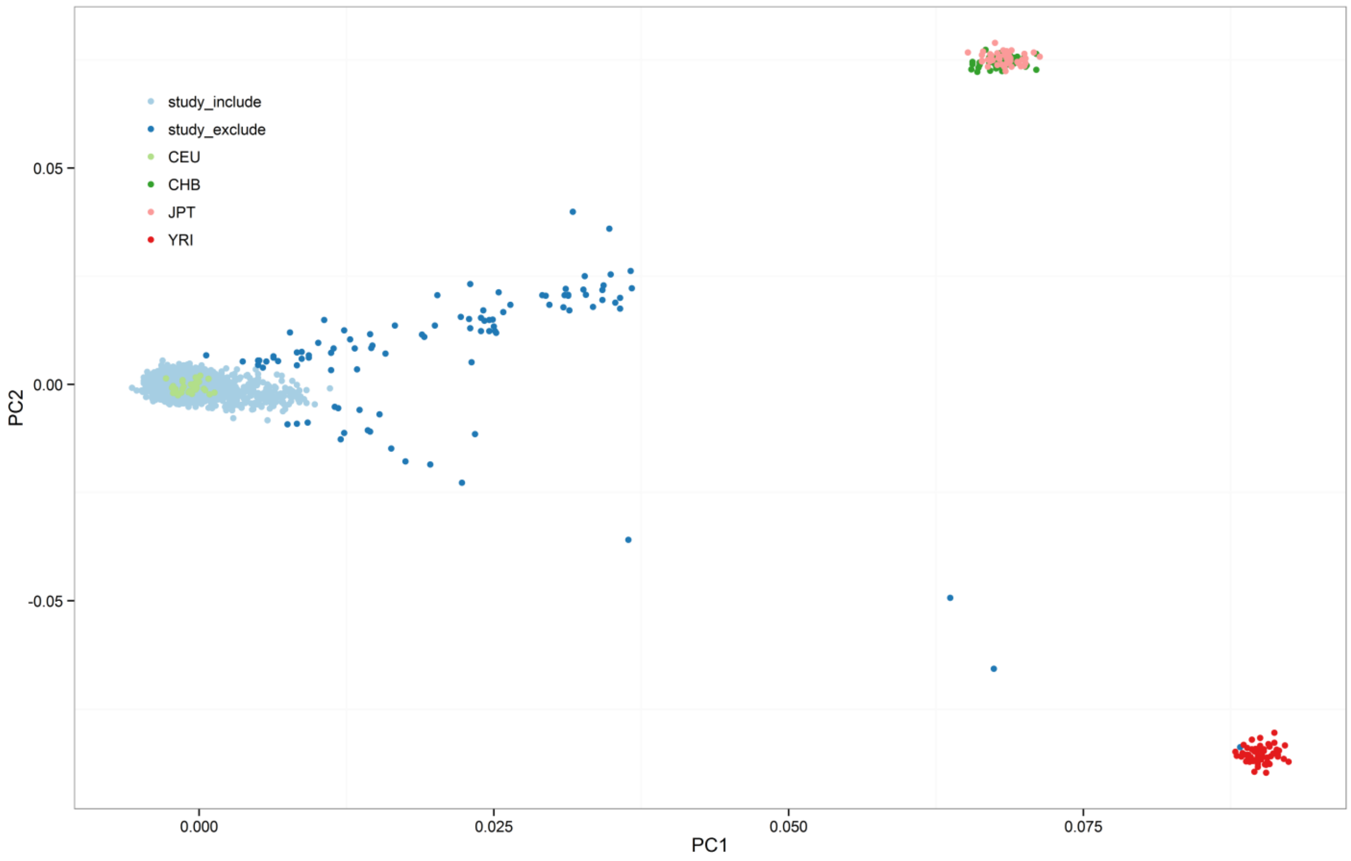

Supplementary Figure 8. Principal component analysis of all study samples against the HapMap population reference panels of CEU, CHB, JPT and YRI datasets. Study samples are coloured blue, those samples excluded based on ancestry are coloured dark blue.

Supplementary Table 1. Summary of sample quality control exclusions by cohort, case-control status and for the study overall.

	Start	<90% CF	Duplicates	Non-study	<98% CF	Autosomal Het.	Fail IBD	Fail HapMap PCA	Remaining
UK cases	1,627	20	12	0	19	7	71	34	1,464
UK controls	4,537	16	0	0	30	13	158	28	4,292
UK controls (UoV)	4,469	27	36	44	35	15	126	9	4,177
Irish cases	312	6	0	0	13	2	32	0	259
Irish controls	478	11	0	0	3	5	2	3	454
Australian cases	278	1	0	0	6	6	3	23	239
Total cases	2,217	27	12	0	38	15	106	57	1,962
Total controls	9,484	54	36	44	68	33	286	40	8,923
Total	11,701	81	48	44	106	48	392	97	10,885

Abbreviations: CF; call frequency, Het; heterozygosity, IBD; identity by descent, PCA; principal component analysis, UoV; University of Virginia.

Supplementary Table 2. Summary of SNP quality control exclusions.

GenomeStudio SNP QC	
Start	196,524
Chromosome Y/MT	1,736
Low cluster separation (< 0.4)	10,076
Low call frequency (< 0.95)	938
Non-polymorphic (all samples)	17,947
Duplicate SNPs	864
call rate (<0.98)	3,041
HWE (p<5x10-7)	1,054
MAF (<0.01)	30,927
hg19 unmappable	67
Total excluded	66,650
Remaining	129,874

Abbreviations: QC; quality control, MT; mitochondria, HWE; Hardy-Weinberg equilibrium, MAF; minor allele frequency.

Supplementary Table 3. Conditional analysis for distinct PsA at known psoriasis susceptibility loci.

Chr.	Gene	PsA SNP	psoriasis SNP	Uncond. P	Cond. P_{single}	Cond. $P_{multiple}$	LRT P	AIC	BIC
1	IL23R	rs12044149	rs11209032	2.25×10^{-15}	1.48×10^{-15}	-	0.33	8246.3/8247.4	8275.2/8283.4
1	IL23R	rs12044149	rs9988642	2.25×10^{-15}	2.40×10^{-14}	-	0.21	8245/8245.4	8273.8/8281.4
2	KCNH7_IFIH1	rs35667974	rs17716942	3.12×10^{-7}	1.75×10^{-7}	2.04×10^{-6}	9.32×10^{-5}	8281/8266.4	8309.8/8309.6
17	PTRF_STAT3_STAT5A_B	rs730086	rs963986	2.74×10^{-5}	2.95×10^{-6}	-	0.005	8288.1/8282	8316.9/8318
17	NOS2	rs4795067	rs28998802	1.94×10^{-7}	5.29×10^{-6}	-	0.003	8281.9/8274.9	8310.7/8310.9
5	IL12B	rs4921482	rs4379175	1.47×10^{-15}	4.79×10^{-5}	5.22×10^{-5}	1.27×10^{-5}	8263.5/8245	8292.4/8288.2
19	TYK2	rs34725611	rs34536443	2.99×10^{-9}	2.88×10^{-6}	0.02	-	-	-
6	EXOC2_IRF4	rs7761186	rs9504361	0.0007088	0.000422	-	-	-	-
5	ERAP1	rs62376445	rs27432	0.0001744	0.0004735	-	-	-	-
6	TAGAP	rs75402062	rs2451258	0.002801	0.000915	-	-	-	-
11	ETS1	rs4936059	rs3802826	0.001475	0.001371	-	-	-	-
9	DDX58	rs1133071	rs11795343	3.36×10^{-5}	0.00294	-	-	-	-
16	PRSS53_FBXL19	rs72793373	rs12445568	0.003249	0.002979	-	-	-	-
7	ELMO1	rs73112675	rs2700987	0.004116	0.005324	-	-	-	-
2	B3GNT2	rs6713082	rs10865331	4.59×10^{-5}	0.008598	-	-	-	-
9	KLF4	rs796754	rs10979182	0.008985	0.008854	-	-	-	-
17	NOS2 (2)	rs8072199	rs28998802	8.86×10^{-5}	0.026	-	-	-	-
2	FLJ16341_REL	rs1306395	rs62149416	2.99×10^{-5}	0.03663	-	-	-	-
10	ZMIZ1	rs1972346	rs1250546	0.008258	0.112	-	-	-	-
6	TNFAIP3	rs610604	rs582757	0.0003245	0.1409	-	-	-	-
5	IL13_IL4	rs848	rs1295685	1.05×10^{-5}	0.1593	-	-	-	-
16	PRM3_SOCS1	rs12928822	rs367569	0.001593	0.1859	-	-	-	-
22	UBE2L3	rs2298428	rs4821124	4.38×10^{-5}	0.3196	-	-	-	-
20	RNF114	rs6063454	rs1056198	2.9×10^{-5}	0.4698	-	-	-	-
2	KCNH7_IFIH1 (2)	rs2111485	rs2111485	6.09×10^{-5}	NA	-	-	-	-
5	IL12B (2)	rs12188300	rs12188300	2.7×10^{-8}	NA	-			
1	IL28RA	rs7552167	rs7552167	1.53×10^{-5}	NA	-	-	-	-
1	LCE3B_LCE3D	rs6693105	rs6677595	0.002892	NA	-	-	-	-
5	TNIP1	rs76956521	rs2233278	4.98×10^{-9}	NA	-	-	-	-
6	TRAF3IP2	rs33980500	rs33980500	2.65×10^{-16}	NA	-	-	-	-
11	RPS6KA4_PRDX5	rs645078	rs645078	0.0008597	NA	-	-	-	-
12	STAT2_IL23A	rs2020854	rs2066819	7.73×10^{-10}	NA	-	-	-	-
14	NFKBIA	rs8016947	rs8016947	9.65×10^{-5}	NA	-	-	-	-
1	SLC45A1_TNFRSF9	rs11121129	rs11121129	0.0009336	NA	-	-	-	-
1	RUNX3	rs7523412	rs7536201	5.42×10^{-6}	NA	-	-	-	-
11	ZC3H12C	rs4561177	rs4561177	0.003782	NA	-	-	-	-
17	CARD14	rs11652075	rs11652075	0.01423	NA	-	-	-	-
18	POL1_STARD6_MBD2	rs602422	rs545979	0.004767	NA	-	-	-	-
19	ILF3_CARM1	rs892085	rs892085	4.02×10^{-5}	NA	-	-	-	-

Abbreviations: Chr; chromosome, Uncond; unconditioned, Cond; condition, LRT; likelihood ratio test, AIC; Akaike Information criterion, BIC; Bayesian Information Criterion.

Supplementary Table 4. Summary of credible SNP set from imputed data at known and novel regions.

Chr	gene	index snp	region size (bp)	region SNPs	credible SNPs	credible SNP set	credible interval	credible interval size (bp)
1	RUNX3	rs7523412	118750	285	52	52 (0.18)	25289734-25305172	15438
1	IL23R	rs12044149	689273	1791	34	34 (0.02)	67600686-67658954	58268
1	DENND1B	rs2477077	724181	1049	37	37 (0.04)	197317238-197812732	495494
2	FLJ16341_REL	rs1306395	1507047	2962	32	32 (0.01)	61068822-61160619	91797
2	B3GNT2	rs6713082	208276	561	22	22 (0.04)	62510388-62560332	49944
2	KCNH7_IFIH1	rs35667974	594932	574	4	4 (0.01)	163110536-163237390	126854
5	P4HA2	rs715285	783424	1644	35	35 (0.02)	131418948-131556203	137255
5	TNIP1	rs76956521	619488	1632	24	24 (0.01)	150464901-150472602	7701
5	IL12B	rs4921482	912295	1373	3	3 (0.002)	158764177-158766022	1845
6	TRAF3IP2	rs33980500	642883	1551	7	7 (0.005)	111580561-111908882	328321
12	STAT2_IL23A	rs2020854	598673	731	121	121 (0.17)	56509918-56753822	243904
14	NFKBIA	rs8016947	683942	2554	480	480 (0.19)	35225483-35887916	662433
17	NOS2	rs4795067	482352	1145	2	2 (0.002)	26106675-26118521	11846
17	PTRF_STAT3_STAT5A_B	rs730086	723206	1085	5	5 (0.005)	40271757-40318734	46977
19	TYK2	rs34725611	373832	1039	5	5 (0.005)	10459969-10477067	17098
20	RNF114	rs6063454	299803	908	132	132 (0.15)	48511644-48659343	147699
22	UBE2L3	rs2298428	168674	447	96	96 (0.21)	21916166-21997591	81425

Credible SNP sets were calculated from imputed data at known and novel loci that, based on posterior probability, have a 99% chance on containing the causal SNP. The table describes the size of the original imputed and the number of SNPs contained within them (region size and region SNPs columns respectively) followed by the number of credible SNPs identified in the Bayesian refinement , the refined interval coordinates and refined interval size (credible SNPs, credible interval and credible interval size columns respectively).

Abbreviations: Chr ;chromosome, bp; base pairs.

Supplementary Table 5. Summary of functional annotation of credible SNP sets using transcript and ENCODE features.

gene	SNPs	exonic	intergenic	intronic	UTR3	UTR5	ncRNA	Tfbs_clusters	Dnase_clusters	h3k4me1	h3k4me3	h3k27ac	h3k9ac
RUNX3	52	1 (0.02)	47 (0.9)	1 (0.02)	0 (0)	0 (0)	0 (0)	17 (0.33)	18 (0.35)	39 (0.75)	17 (0.33)	23 (0.44)	15 (0.29)
IL23R	34	0 (0)	16 (0.47)	15 (0.44)	0 (0)	0 (0)	0 (0)	9 (0.26)	3 (0.09)	9 (0.26)	4 (0.12)	7 (0.21)	0 (0)
DENND1B	37	0 (0)	9 (0.24)	23 (0.62)	1 (0.03)	0 (0)	0 (0)	5 (0.14)	5 (0.14)	1 (0.03)	1 (0.03)	1 (0.03)	1 (0.03)
FLJ16341_REL	32	0 (0)	7 (0.22)	0 (0)	0 (0)	0 (0)	21 (0.66)	5 (0.16)	7 (0.22)	15 (0.47)	5 (0.16)	5 (0.16)	8 (0.25)
B3GNT2	22	0 (0)	22 (1)	0 (0)	0 (0)	0 (0)	0 (0)	8 (0.36)	10 (0.45)	22 (1)	3 (0.14)	11 (0.5)	12 (0.55)
KCNH7_IFIH1	4	1 (0.25)	2 (0.5)	1 (0.25)	0 (0)	0 (0)	0 (0)	0 (0)	0 (0)	0 (0)	0 (0)	0 (0)	0 (0)
P4HA2	35	0 (0)	16 (0.46)	17 (0.49)	0 (0)	0 (0)	2 (0.06)	5 (0.14)	8 (0.23)	10 (0.29)	4 (0.11)	4 (0.11)	0 (0)
TNIP1	24	0 (0)	19 (0.79)	5 (0.21)	0 (0)	0 (0)	0 (0)	11 (0.46)	8 (0.33)	24 (1)	24 (1)	24 (1)	7 (0.29)
IL12B	3	0 (0)	0 (0)	0 (0)	0 (0)	0 (0)	3 (1)	0 (0)	1 (0.33)	3 (1)	0 (0)	0 (0)	0 (0)
TRAF3IP2	7	1 (0.14)	0 (0)	4 (0.57)	0 (0)	1 (0.14)	1 (0.14)	2 (0.29)	2 (0.29)	4 (0.57)	2 (0.29)	3 (0.43)	1 (0.14)
STAT2_IL23A	121	8 (0.07)	26 (0.21)	68 (0.56)	5 (0.04)	1 (0.01)	0 (0)	29 (0.24)	19 (0.16)	22 (0.18)	31 (0.26)	28 (0.23)	23 (0.19)
NFKBIA	480	3 (0.01)	157 (0.33)	252 (0.52)	1 (0)	4 (0.01)	30 (0.06)	94 (0.2)	66 (0.14)	106 (0.22)	98 (0.2)	62 (0.13)	59 (0.12)
NOS2	2	0 (0)	0 (0)	2 (1)	0 (0)	0 (0)	0 (0)	1 (0.5)	1 (0.5)	0 (0)	0 (0)	0 (0)	0 (0)
PTRF_STAT3_STAT5A_B	5	0 (0)	0 (0)	4 (0.8)	0 (0)	0 (0)	0 (0)	1 (0.2)	1 (0.2)	1 (0.2)	1 (0.2)	0 (0)	0 (0)
TYK2	5	1 (0.2)	1 (0.2)	3 (0.6)	0 (0)	0 (0)	0 (0)	1 (0.2)	2 (0.4)	0 (0)	0 (0)	0 (0)	0 (0)
RNF114	132	2 (0.02)	86 (0.65)	22 (0.17)	4 (0.03)	0 (0)	1 (0.01)	35 (0.27)	33 (0.25)	14 (0.11)	13 (0.1)	13 (0.1)	7 (0.05)
UBE2L3	96	1 (0.01)	5 (0.05)	76 (0.79)	3 (0.03)	0 (0)	0 (0)	55 (0.57)	23 (0.24)	33 (0.34)	27 (0.28)	10 (0.1)	16 (0.17)

Abbreviations: UTR; untranslated region, Tfbs; transcription factor binding site.

Supplementary Table 6. Summary of independent effects identified at three loci.

SNP	Chr.	Position (bp)	Notable genes	Conditional SNP	Risk/ non-risk allele	RAF (case)	RAF (control)	P value	LD with index SNP (r^2/D')
rs12188300	5	158829527	*IL12B*	rs4921482	T/A	0.13	0.1	2.70E-08	0.007/0.354
rs2111485	2	163110536	*IFIH1*	rs35667974	G/A	0.65	0.61	6.09E-05	0.031/1.000
rs8072199	17	26116848	*NOS2*	rs4795067	C/T	0.59	0.55	8.86E-05	0.006/0.094

Abbreviations: Chr; chromosome, bp; base position, RAF; risk allele frequency, LD; linkage disequilibrium.

Supplementary Table 7. Enrichment of 20 associated variants at H3K4me3 chromatin marks in 34 cell and tissue types.

Tissues	psa_1e-04_score
CD8_Memory_Primary_Cells	0.0016
Duodenum_Mucosa	0.0371
Adult_Liver	0.0563
CD4_Naive_Primary_Cells	0.0702
Colonic_Mucosa	0.0959
Skeletal_Muscle	0.1173
Treg_Primary_Cells	0.1305
Rectal_Smooth_Muscle	0.1416
Adipose_Nuclei	0.1737
Bone_Marrow_Derived_Mesenchymal_Stem_Cell_Cultured_Cells	0.2086
Adult_Kidney	0.2175
CD19_Primary_Cells	0.2555
Adipose_Derived_Mesenchymal_Stem_Cell_Cultured_Cells	0.2917
Mesenchymal_Stem_Cell_Derived_Adipocyte_Cultured_Cells	0.2935
CD4_Memory_Primary_Cells	0.3738
CD3_Primary_Cells	0.3756
CD34_Primary_Cells	0.3894
CD8_Naive_Primary_Cells	0.4114
Muscle_Satellite_Cultured_Cells	0.4915
Brain_Substantia_Nigra	0.6134
Chondrocytes_from_Bone_Marrow_Derived_Mesenchymal_Stem_Cell_Cultured_Cells	0.6253
Mobilized_CD34_Primary_Cells	0.6734
Stomach_Mucosa	0.7164
Pancreatic_Islets	0.7185
Duodenum_Smooth_Muscle	0.7306
CD34_Cultured_Cells	0.7395
Brain_Mid_Frontal_Lobe	0.8586
Stomach_Smooth_Muscle	0.8815
Brain_Cingulate_Gyrus	0.8928
Rectal_Mucosa	0.9132
Colon_Smooth_Muscle	0.9246
Brain_Inferior_Temporal_Lobe	0.9356
Brain_Anterior_Caudate	0.9476
Brain_Hippocampus_Middle	0.9482

Supplementary Table 8: SNP specificity scores for the enrichment of 20 associated variants at H3K4me3 chromatin marks in CD8^{+} memory primary T-cells.

Index SNP	Best SNP	Score	Distance
rs715285	rs10065787	0.859663	84
rs4921482	rs4921482	0.655801	500
rs1306395	rs842638	0.503709	1979
rs2020854	rs80317430	0.297581	7
rs892085	rs4804520	0.208939	563
rs76956521	rs76462670	0.089165	143
rs7552167	rs4090311	0.088382	577
rs2298428	rs2266959	0.068183	167
rs12044149	rs12044149	0.041618	101
rs7523412	rs6672420	0.015026	392
rs1133071	rs1133071	0	NA
rs6713082	rs6713082	0	NA
rs33980500	rs33980500	0	NA
rs34725611	rs34725611	0	NA
rs848	rs848	0	NA
rs6063454	rs6063454	0	NA
rs4795067	rs4795067	0	NA
rs984971	rs984971	0	NA
rs2477077	rs2477077	0	NA
rs8016947	rs8016947	0	NA

Supplementary Table 9: Summary statistics for validation of three independent effects to HLA genes in 572 cases and 888 controls.

Gene	Position	Allele/residue	Omnibus p-value	A1/A2	p-value†	Minor allele freq.		Odds Ratio†	CI†
						Cases	Controls		
HLA-C	-	*0602	$1.59x10^{-14}$	P/A	-	0.22	0.10	3.11	2.30:4.24
HLA-B	67	Cysteine	$1.89x10^{-12}$	P/A	$4.09x10^{-14}$	0.20	0.12	2.85	2.18:3.75
		Phenylalanine		P/A	0.34	0.22	0.28	1.19	0.89:1.41
		Methionine		P/A	0.97	0.09	0.05	0.99	0.66:1.50
		Tyrosine		P/A	0.43	0.11	0.16	0.89	0.68:1.18
		Serine		P/A	ref	0.37	0.40	ref	ref
HLA-A	107	Glycine	$3.3x10^{-9}$	Glycine/Tryptophan	-	0.38	0.26	1.74	1.44:2.12

Abbreviations: A1: allele 1, A2; allele2, P; present, A; absent, freq; frequency, CI; confidence intervals. †Calculated in regression model containing *HLA-C*0602, HLA-B* amino acid position 67 and *HLA-A* amino acid position 107.
